# The Impact of *Oulema melanopus*—Associated Bacteria on the Wheat Defense Response to the Feeding of Their Insect Hosts

**DOI:** 10.3390/cells11152342

**Published:** 2022-07-29

**Authors:** Beata Wielkopolan, Patryk Frąckowiak, Przemysław Wieczorek, Aleksandra Obrępalska-Stęplowska

**Affiliations:** 1 Department of Monitoring and Signaling of Agrophages, Institute of Plant Protection—A National Research Institute, 60-318 Poznań, Poland; b.wielkopolan@iorpib.poznan.pl; 2 Department of Molecular Biology and Biotechnology, Institute of Plant Protection—A National Research Institute, 60-318 Poznań, Poland; p.frackowiak@iorpib.poznan.pl (P.F.); p.wieczorek@iorpib.poznan.pl (P.W.)

**Keywords:** microbiome, beetles, Coleoptera, plant response, wheat, plant-insect-bacteria interactions

## Abstract

Wheat production is threatened by the destructive effects of numerous pests, including *Oulema melanopus* (cereal leaf beetle, CLB). Both adults and larvae of CLB damage grain crops, but the target of insecticide treatments are the larvae. Insect-associated bacteria are important for many of the insects’ life processes and may also modulate plant defense responses to feeding of their insect host. The aim of our study was to elucidate the early wheat plants’ reaction to this herbivore feeding and to disclose the CLB-associated bacteria modulation of the wheat-insect interactions. Transcriptome analyses were performed for the leaves wounded mechanically and by feeding of the CLB larvae as well as for the distal leaves to study both, the plant’s local and systemic response. Comparative transcriptome analysis indicated that 24 h after the plant treatment, a much larger number of up-regulated DEGs in damaged leaves was noted, especially those on which larvae were fed. It may suggest that at the analysed time point, the local response was stronger than the systemic one. In the leaves on which larvae with natural bacterial flora were fed (local response), the number of up- and down-regulated differentially expressed genes (DEGs) was 7136 and 7411, respectively, in comparison to the dataset obtained for the leaves wounded by larvae with a reduced number of bacteria. In the distal leaves, 3015 up- and 2372 down-regulated DEGs were noted. CLB-associated bacteria were found to affect many aspects of the physiology of wheat plants, especially in wounded leaves, including the expression of genes related to primary metabolism, phytohormone signaling and photosynthesis. We also observed that CLB-associated bacteria mitigated numerous anti-herbivore processes and pathways associated with the synthesis of metabolites and proteins, potentially harmful to the insects. The bacteria also reversed the expression of some genes involved, inter alia, in the phosphorylation of proteins, oxidative stress, cell wall organization, and biogenesis. Understanding the role of CLB-associated bacteria in the plant’s defense response will be important to the fields of pest control and herbivore and its host ecology and evolution.

## 1. Introduction

Wheat (*Triticum aestivum* L.) is one of the most widely grown staple crops, providing a major source of energy and dietary fiber for humans [1]. According to the Food and Agriculture Organization of the United Nations (FAOSTAT), the global production of wheat reached 761 million tonnes (219 million ha of harvested area) in 2020. In the European Union alone, it was around 126.7 million tonnes (22.8 million ha of harvested area) (FAOSTAT 2021). Wheat production is, however, affected by numerous pathogens and pests, among them the cereal leaf beetle (CLB, *Oulema melanopus*, Chrysomelidae), one of the major insect pests of this crop species. Both adults and larvae of CLB damage grain crops by feeding on the leaves, which results in reduced quality and quantity of the obtained crop yield. Additionally, like many other herbivorous insect pests, CLB might transmit pathogens, which can further enhance crop losses [2,3]. The larvae of CLB are considered the most damaging stage, and thus are the target of insecticide treatments [4].

Plants have evolved a wide range of defense mechanisms to effectively protect themselves against various types of invaders, including viruses, bacteria, fungi, or herbivorous insects [5], which attack simultaneously or sequentially. The attacks result in changes in the primary metabolism of the plant [6] as well as the expression levels of defense-related genes (like *pathogenesis-related* genes, *PR*) and an increase in the levels of different phytohormones, such as salicylic acid (SA) or jasmonic acid (JA). Plants may also activate systemic responses, such as systemic acquired response (SAR) or induced systemic response (ISR), that are important in managing biotic stresses [7].

Plants recognize insect pests by their feeding guilds, feeding patterns, and feeding positions [8]. Insects produce herbivore-associated molecular patterns, which could arise among others from insects’ oral secretions (regurgitants), saliva, frass, or herbivore-associated endosymbionts [9]. Salivary components are highly complex and, when applied to mechanically wounded tissues, can have an impact on gene expression, by either inducing or repressing the level of some transcripts [10,11]. Generally, herbivorous insects are associated with a wide variety of microorganisms, including bacteria. The CLB is known to be associated with a whole range of bacteria (such as those belonging to the *Acinetobacter*, *Stenotrophomonas*, *Streptococcus*, *Serratia*, *Moraxella*, *Lactobacillus*, *Pseudomonas*, and *Pantoea* genus), and their composition can be influenced by various factors, including diet [12]. However, regardless of the variables analysed (developmental stage, cereal host species and locations from which insects were collected), at least 90% of the tested CLB insects were associated with bacteria belonging to the genera *Wolbachia* and *Rickettsia* [12].

It has been indicated that bacteria affect many of the insects’ life processes, such as growth, digestion, or adaptation to feed on new plant species. Moreover, insect-associated bacteria can be engaged in manipulating plant responses to herbivore feeding [13,14,15,16,17], leading to, e.g., suppression of the induction of plant defenses [18]. Recently, much effort has been made to understand the role of insect-associated bacteria in altering plant immune responses to insect attacks [13,16,19]. For instance, it has been shown that CLB-associated bacteria suppress the expression of the gene encoding serine protease inhibitor, which probably can inhibit the proteolytic activity of insect enzymes [19]. In turn, Chung et al. [16] have indicated that antibiotics-untreated larvae of the Colorado potato beetle (CPB, *Leptinotarsa decemlineata*, Chrysomelidae) suppressed JA-responsive anti-herbivore defenses and activated anti-microbial responses through the induction of an SA-dependent signaling pathway, which resulted in improved larval growth.

Although CLB is one of the most serious pests of cereals, not much is known about the plants’ response to the damage it causes and about the way the bacteria associated with CLB affect the plants’ defense during the feeding of their insect host. Our aim was to systematically characterize the wheat’s defense response to CLB larvae feeding and to study the effect of larval-associated bacteria on the plant’s response. Transcriptome analyses were performed for the leaves wounded by the larvae as well as for the distal leaves to study both the early plant’s local and systemic defense response.

Exploration of the effects of the bacteria associated with CLB larvae can provide a better understanding of the three-way interactions between wheat, CLB, and its microbiome. The results can give answers to how much the presence of CLB larvae-associated bacteria affects the defense responses and metabolism of the insect-damaged plants and may contribute to the development of new methods of control of this pest and to the expansion of knowledge within the framework of plant host and herbivorous insect ecology and evolution. To the best of our knowledge, this is the first study of the wheat transcriptome changes in response to CLB larvae feeding, including the role of larvae-related bacteria.

## 2. Materials and Methods

### 2.1. Plant Material and Insects

Winter wheat of the Arkadia variety (from the DANKO company, Choryń, Poland) was used in the study. The selected plants were grown for a month under the following conditions: 22 °C during the day and 18 °C at the night, humidity ~45–55%, 16L/8D, prior to the experiment. The plants were divided into four groups according to the variant of treatment: (a) unwounded, (b) mechanically damaged (the CLB larvae feeding was imitated by scraping the upper leaf tissue into the lower epidermis with a sterile needle; the size of damage was similar to that caused by larvae feeding), (c) wounded by the antibiotics-treated CLB larvae (reduced number of insect-associated bacteria), and (d) wounded by the antibiotics-untreated CLB larvae (with natural intestinal microflora). The experiment was carried out in the Research Centre of Quarantine, Invasive and Genetically Modified Organism, the Institute of Plant Protection—a National Research Institute in Poznań (Poland). Plant material, for all the tested conditions, was collected 24 h after plant treatments, frozen in liquid nitrogen, and stored at −80 °C. For each plant, two leaves were harvested—the damaged one (mechanically or by CLB larvae feeding) and a distal leaf. Two leaves from undamaged plants were also collected from the positions corresponding to those of the damaged and distal leaves. For each treatment, three biological replicates were made.

CLB larvae were collected in Winna Góra (52°12′17″ N 17°26′48″ E) from the field. Larvae employed in each plant treatment were of the same instar and similar in size (3 mm). Before applying the larvae to the wheat leaves, some of them were treated with a mixture of antibiotics (0.01 g neomycin sulfate, 0.05 g chlortetracycline, 0.003 g streptomycin, and 0.04 g methylparaben in 50 mL of ultrapure Milli-Q water (MQ water)) to reduce the number of insect-associated bacteria. The mixture of antibiotics was applied to wheat leaves, which had previously been disinfected in 70% ethanol and rinsed twice in sterile MQ water. The leaves were placed on a 1% agarose medium (Petri dishes) to retain moisture. The larvae fed on the wheat leaves for 48 h, so that all the plant material was eaten. Next, the larvae were applied to the wheat plants prepared for the main experiment.

### 2.2. Extraction and Purification of RNA

Total RNA was extracted from the collected plant material using the RNeasy Plant Mini Kit (Qiagen, Wroclaw, Poland). For each treatment, three biological replicates were prepared. RNA concentration was determined using the fluorometric-based method on a Qubit 4 Fluorometer (Thermo Scientific, Lenexa, KS, USA). Furthermore, the RNA integrity was assessed by the 2100 Bioanalyzer (Agilent, Santa Clara, CA, USA).

### 2.3. The RNA-Sequencing and Data Analysis

The purified total RNA samples from wheat plants were used to prepare cDNA libraries. The preparation of libraries was carried out by CeGaT GmbH (Tübingen, Germany) using TruSeq Stranded RNA (Illumina, San Diego, CA, USA) with the RiboZero Plant kit according to the manufacturer’s protocol. Demultiplexing of the sequencing reads was performed with Illumina bcl2fastq (2.20, San Diego, CA, USA, 30 August 2017). Adapters were trimmed with Skewer (Version 0.2.2, Beijing, China, 4 April 2016) [20]. The sequence data were submitted to the SRA database under the BioProject ID PRJNA839232 (https://submit.ncbi.nlm.nih.gov/about/sra/, accessed on 20 January 2021). The quality check of the obtained raw data was performed using FastQC software in the OmicsBox application (OmicsBox—Bioinformatics made easy, BioBam Bioinformatics (Version 1.2.4, Valencia, Spain, 3 March 2019, www.biobam.com/omicsbox, accessed on 20 January 2021) [21]. The filtered clean reads from each sample were aligned to the reference genome of *T. aestivum* available on the Ensembl Plants database (http://ftp.ensemblgenomes.org/pub/plants/release-52/fasta/triticum_aestivum/dna_index/, ‘Triticum_aestivum.IWGSC.dna.toplevel.fa’, accessed on 20 January 2021). Mapping and subsequent analyses were carried out using the OmicsBox software [22,23,24]. The count table obtained after the mapping process was filtered using the edgeR packet in the R environment (Version 3.6.2, Vienna, Austria, 2021) [24].

The differential gene expression analysis was carried out by using the OmicsBox [24] software. To assess the effect of larvae feeding on the plant response, the gene expressions in the treated plants (plants damaged mechanically, damaged by larvae with natural intestinal microflora, or with a reduced bacterial flora) were compared to those in the unwounded plants (control plants). To answer the question of the role of bacteria associated with CLB larvae in the wheat defense response, the results obtained for the plants exposed to the feeding of larvae with natural bacterial flora were compared to those of the plants exposed to the feeding of larvae whose number of bacteria had been reduced. The number of clean reads that mapped to each annotated transcript after RNA-sequencing (RNA-seq) analysis was calculated using the HTSeq algorithm followed by normalization according to the TMM (Trimmed Mean of M values) method. Further data analysis was carried out using the GLM (Quasi-likelihood F test). The differentially expressed genes (DEGs) were filtered by *p*-value ≤ 0.05 and log2 fold change −1 ≥ (FC) ≥ 1. The annotation file for *T. aestivum* genes was downloaded from the BioMart Ensembl database. To obtain functional annotation and identify putative biological pathways of statistically significant DEGs, the NCBI Gene Ontology (GO) [25] and the Kyoto Encyclopedia of Genes and Genomes (KEGG) [26] databases were used, and all procedures were performed in the OmicsBox software. The *p*-value < 0.05 was used as a threshold to define significantly enriched GO terms (Fisher’s exact test) and KEGG pathways. The OmicsBox software and R software with GOplot [27] and ggplot2 (https://www.rdocumentation.org/packages/ggplot2/versions/3.3.3, Springer-Verlag, New York, NY, USA, accessed on 30 December 2020) packages were used to specify the most enriched GO terms.

### 2.4. Validation of RNA-Sequencing Results by Quantitative Real-Time PCR Analysis

To validate the reliability of the sequencing results, the expression levels of seven transcripts that were significantly altered in RNA-sequencing analysis in the plants damaged by larvae-associated bacteria were selected for RT-qPCR. RNA was isolated from the same leaves from which RNA was isolated for sequencing. Total RNA (2 µg) was reverse transcribed using a Maxima First cDNA synthesis Kit with DNase (Thermo Fisher, Lenexa, KS, USA) according to the manufacturer’s instructions. RT-qPCR reactions were performed in a total volume of 10 µL, containing 1 µL of cDNA, 0.5 µM of reverse and forward primers, 3 µL of ultrapure Milli Q water, and 5 µL of iTag^TM^ Syber Green 2× Master Mix (Bio-Rad, Hercules, CA, USA). *Ta54227*, a gene encoding cell division protein [28], was used as a reference gene in the experiment (three reference genes were tested and *Ta54227* was the most stable). The samples were run on a LightCycler^®^ 480 (Roche Applied Science, Mannheim, Germany) according to the following procedure: Five minutes after initial denaturation at 95 °C, the regime performed 45 cycles of amplification: 10 s at 95 °C, 20 s at 55 °C, and 30 s at 72 °C. The melting curve was prepared after the last RT-qPCR cycle. Three biological and technical replicates were set for each sample, and ultrapure Milli Q water was used as the blank control. All the primer sequences were indicated in the Supplementary Table S1. The RT-qPCR data were analysed using GenEx software version 6. Because some of the obtained results did not meet the assumptions of normal distribution, the data were analysed using the non-parametric Mann–Whitney test for *p*-value < 0.05.

## 3. Results

### 3.1. Transcriptomic Profile of the Wheat Plants Subjected to the Treatments Applied

To analyse the defense response of wheat plants to each of the treatments, the transcriptomic profiles of mechanically damaged plants (A), and plants on which CLB larvae with natural bacterial flora (B), or with a reduced number of bacteria (C) were feeding, were compared to the transcriptome datasets obtained for the undamaged plants (as controls). The appropriate controls were prepared for the damaged (local response) and distal leaves (systemic response), respectively. The results are presented below.

#### 3.1.1. Distribution of DEGs in Wheat Plants Damaged Mechanically and by CLB Larvae Feeding

**Figure 1:**
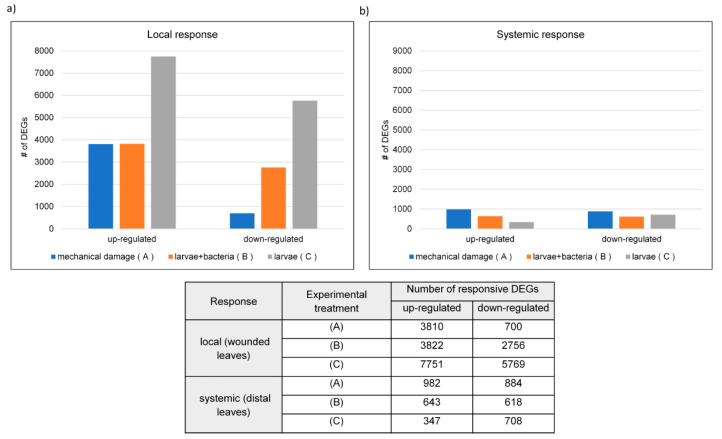
Distribution of up- and down-regulated differentially expressed genes (DEGs) in wheat plants subjected to mechanical damage (A), feeding of CLB larvae with natural bacterial flora (B), with a reduced number of bacteria (C), for local (**a**) and systemic plant response (**b**), respectively.

In wounded leaves, for all experimental conditions, the number of up-regulated DEGs was higher than down-regulated ones. The difference between the number of up- and down-regulated DEGs was higher in mechanically wounded plants (A, 62% up- and 38% down-regulated). In wheat leaves exposed to CLB larvae with natural bacterial flora (B), 6578 DEGs were reported, of which 58% were up-regulated while 42% were down-regulated. The largest number of DEGs (13,520) was observed for plants exposed to the feeding of the CLB larvae with a reduced number of bacteria (C), of which 57% were up-regulated and 43% were down-regulated (Figure 1a).

The plant response in the distal leaves resulted in a lower number of observed DEGs, in comparison to those obtained for the wounded leaves. More DEGs were up- (982) and down-regulated (884) in response to mechanical tissue injury (A) than in response to the other two plant treatments (B and C). A total of 1261 DEGs were related to the wounding caused by CLB larvae with natural intestinal microflora (B), of which 51% were up-regulated and 49% down-regulated. The difference between the numbers of up- and down-regulated DEGs was the greatest in the plants exposed to the antibiotics-treated larvae (C). In these plants, 1055 DEGs were reported, among which 33% were up-regulated while 67% were down-regulated (Figure 1b).

A much larger number of up-regulated DEGs in damaged leaves, especially those damaged by the larvae, may suggest that at the analysed time point the local response was stronger than the systemic one and that in the plants damaged by larvae, particularly in those exposed to antibiotics-treated larvae, greater changes in the expression levels of genes associated with metabolic pathways took place. The differences in DEGs distribution in the leaves exposed to the antibiotics-treated and non-treated larvae may indicate that the plant’s response was significantly influenced by the bacteria associated with the insect. The differences in DEGs distribution between the mechanically damaged leaves (A) and those wounded by the larvae (B, C) may suggest that the plants were able to distinguish between the mechanical tissue injury and the insect damage.

#### 3.1.2. Distribution of Common and Unique DEGs

**Figure 2:**
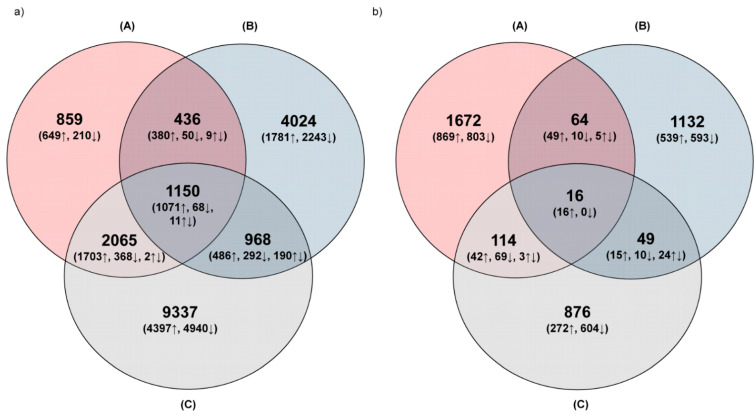
Venn diagrams showing the distribution of unique and common differentially expressed genes in (**a**) wounded and (**b**) distal wheat leaves, in response to mechanical damage (**A**), feeding of CLB larvae with natural bacterial flora (**B**), and feeding of CLB larvae with a reduced number of bacteria (**C**). ↑ Upper index arrows—up-regulated DEGs; ↓ Lower index arrows—down-regulated DEGs; ↑↓ Both index arrows—different expression profiles of common DEGs.

In the wounded wheat leaves of the plants subjected to all variants of treatment, the number of common DEGs was 1150 (Figure 2a and Table S2). The leaves of the plants subjected to mechanical tissue injury (A) and feeding of CLB larvae with a reduced number of bacteria (C) shared the largest number of common DEGs (2065). The plants exposed to CLB larvae with natural bacterial flora (B) and a reduced number of bacteria (C) shared 968 DEGs (Figure 2a and Table S2).

The largest number of unique DEGs was observed for the plants exposed to the antibiotics-treated larvae (C; 9337), and lower for the plants wounded by antibiotics-untreated CLB (B; 4024) (Figure 2a and Table S3). The overall number of unique DEGs in the plants subjected to mechanical damage (A; 859) was significantly lower, compared to that for the other two variants of plant treatments. In the plants exposed to CLB larvae (B and C treatments), the number of down-regulated DEGs was higher than that of up-regulated DEGs, in contrast to their relationship to the mechanically damaged plants (A) (Figure 2a).

In distal leaves, only 16 DEGs were common in the plants subjected to all variants of treatments (Figure 2b and Table S2). The distal leaves from the plants exposed to CLB larvae, treated (C) or untreated with antibiotics (B), shared 49 genes. The number of unique DEGs in the distal leaves of the plants exposed to CLB larvae feeding was lower (B-1132 and C-876, respectively) in comparison to that in the wounded leaves (the local response) (Figure 2 and Table S3).

#### 3.1.3. Gene Ontology Analysis of Common and Unique DEGs for Each Variant of Plant Treatment

In terms of local response data, enrichment analysis of GO terms within the biological processes category (BP) revealed that the majority of the common up-regulated DEGs induced by all of the plant treatment variants (A + B + C) were associated with the following GO terms: lipid biosynthetic process (70 DEGs), lipid transport (22 DEGs), cellulose biosynthetic process (18 DEGs), glycolytic process (14 DEGs), defense response to a bacterium (14 DEGs), and plant-type secondary cell wall biogenesis (14 DEGs) (Table S4), while the down-regulated DEGs were related to intermembrane lipid transfer (2 DEGs). The up-regulated DEGs induced by CLB larvae feeding (B + C) were related to the response to heat (10 DEGs) and glycolytic process (8 DEGs), while the common down-regulated groups of DEGs were assigned to protein dephosphorylation (6 DEGs), cell surface receptor signaling pathway (4 DEGs) and positive regulation of transcription, and DNA templated (4 DEGs). A small number of common DEGs within the BP category for mechanically damaged and larvae-damaged plants with natural bacterial flora (A + B) are given in Table S4. In contrast, for the mechanically damaged plants and those exposed to larvae with a reduced number of bacteria (A + C), a large number of the same up-regulated DEGs were associated, among others, with processes related to the response to oxidative stress (48 DEGs), hydrogen peroxide catabolic process (40 DEGs), fatty acid biosynthetic process (24 DEGs), or L-phenylalanine catabolic process (18 DEGs) (Table S4).

As regards the unique DEGs in the mechanically damaged plants (A), their highest number was noted for the following GO terms: systemic acquired resistance (9 up-regulated DEGs) and regulation of transcription, DNA-templated (17 down-regulated DEGs) (Table S5). In the leaves wounded by CLB larvae with natural bacteria flora (B), the most abundant unique, up-regulated DEGs were related to protein dephosphorylation (20 DEGs), while the down-regulated DEGs were mainly involved in protein phosphorylation (262 DEGs) and cell wall organization (23 DEGs) (Table S5). In the wheat leaves exposed to antibiotics-treated larvae (C), a large number of unique, up-regulated DEGs were recorded for the GO terms: cell redox homeostasis (41 DEGs), glycolytic process (31 DEGs), fatty acid biosynthetic process (31 DEGs), and ATP synthesis coupled proton transport (30 DEGs), whereas the down-regulated DEGs were mainly associated with the recognition of pollen (29 DEGs) and protein glycosylation (25 DEGs) (Table S5).

In distal leaves, the common up-regulated DEGs were mainly associated with protein phosphorylation (4 DEGs) (Table S6). The GO terms of the most abundant unique DEGs for three analysed variants of plant treatments for systemic response are shown in Table S7. For instance, a large number of unique up-regulated DEGs in the mechanically damaged plants (A) were involved in phosphorylation (70 DEGs) and fatty acid biosynthetic processes (22 DEGs).

### 3.2. The Effect of Larval-Associated Bacteria on the Plant Defense Response

To determine the effect of larval-associated bacteria on the plant defense response, the RNA-seq results for the plants exposed to the insects with natural intestinal microflora were compared to those obtained for the plants wounded by larvae with a reduced number of bacteria (as controls). The results are presented below.

#### 3.2.1. Gene Ontology Overview of Larval-Associated Bacteria-Responsive DEGs

In the leaves on which larvae with natural bacterial flora were fed (local response), the number of up-and down-regulated DEGs was 7136 and 7411, respectively, in comparison to the dataset obtained for the leaves wounded by antibiotics-treated larvae. In the distal leaves, 3015 up- and 2372 down-regulated DEGs were recorded (Figure S1).

**Figure 3:**
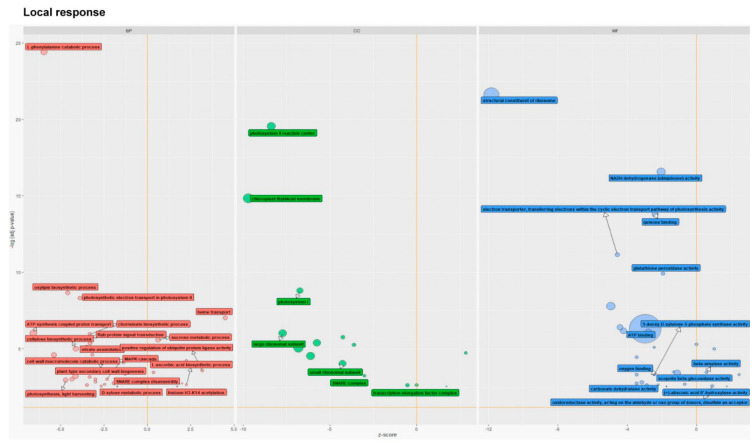
GO functional enrichment analysis of differentially expressed genes in the leaves wounded (local response) by CLB larvae with natural bacterial flora (compared to data obtained for the leaves damaged by CLB larvae with a reduced number of bacteria, as controls). The z-score was assigned to the *x*-axis and the negative logarithm of the adjusted *p*-value to the *y*-axis (the higher the more significant). The area of the displayed circles is proportional to the number of genes assigned to the term, and the color corresponds to the three categories: biological process (BP, red), cellular component (CC, green), and molecular function (MF, blue). Selected circles are labelled with the GO term name.

Enrichment analysis of GO terms within the categories of BP, molecular function (MF), and cellular component (CC) was performed to determine which of them were significant in the plants damaged by CLB larvae with natural intestinal microflora. As we found, the most significant changes in defense-related processes occurred in the wounded wheat leaves. For instance, the GO terms related to, among others, the L-phenylalanine catabolic process, oxylipin biosynthetic process, or photosynthetic electron function in photosystem II, were the most significant. It was observed that at the analysed time point, when the antibiotics-untreated larvae were fed on the leaf, most processes were down-regulated, among others, the above-mentioned processes, the cell wall macromolecule catabolic and chorismate biosynthetic process (Figure 3). In the MF category, the GO terms related to a structural constituent of the ribosome, NADH dehydrogenase (ubiquinone) activity, quinone binding, electron transporter, transferring electron within the cyclic electron transport pathway of photosynthesis activity, and glutathione peroxidase activity were the most significant in the leaves exposed to CLB larvae with the natural bacterial flora. Most of the GO terms within the MF category were down-regulated, including scopolin beta-glucosidase activity, oxygen binding, 1-deoxy-D-xylulose-5-phosphate synthase activity, and carbonate dehydratase activity. The CLB larvae-associated bacteria were found to induce the up-regulation of GO terms related to beta-amylase activity. The GO terms, ATP binding and structural constituents of the ribosome, were associated with the largest number of DEGs. In the CC category, the most significant GO terms were photosystem II reaction center, chloroplast thylakoid membrane, and photosystem I. Most of these GO terms were down-regulated (Figure 3).

**Figure 4:**
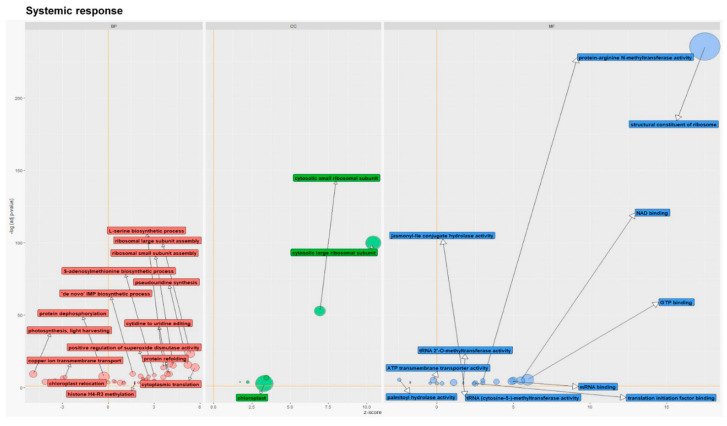
GO functional enrichment analysis of differentially expressed genes in wheat distal leaves (systemic response) to the feeding of CLB larvae with natural bacterial flora (compared to that in the leaves wounded by CLB larvae with a reduced number of bacteria, as controls). The z-score was assigned to the *x*-axis and the negative logarithm of the adjusted *p*-value to the *y*-axis (the higher the more significant). The area of the displayed circles is proportional to the number of genes assigned to the term, and the colour corresponds to the three categories: biological process (BP, red), cellular component (CC, green), and molecular function (MF, blue). Selected circles were labelled with the GO term name.

In distal leaves, the most significant response to feeding of CLB larvae with natural bacterial flora was related to the processes associated with ribosomal large and small subunit assembly, maturation of LSU-rRNA from tricistronic rRNA transcript (SS-rRNA, 5.8S rRNA, LSU-rRNA), pseudouridine synthesis, cytoplasmic translation, and L-serine biosynthetic process (Figure 4). Most of the processes, including those mentioned above, were up-regulated. The presence of bacteria associated with the CLB larvae correlated with the down-regulation of processes linked to photosynthesis, light-harvesting, or copper ion transmembrane transport. Most of the GO terms from the MF category were up-regulated. The GO term-structural constituents of the ribosome, were the most significant upon feeding of antibiotics-untreated CLB larvae and were associated with the highest number of DEGs (mainly up-regulated ones) (Figure 4). Within the CC category, the GO terms related to cytosolic large, and small ribosomal subunits were the most significant in the plants exposed to CLB larvae with natural bacterial flora. The highest number of DEGs was related to the following GO terms: the chloroplast, cytosolic large, and small ribosomal subunits (Figure 4).

As shown by the obtained results, 24 h after the initiation of larvae feeding, the microbiome significantly shaped the plant’s local response (the insects’ feeding sites), also mitigating its defense systems (down-regulation of numerous processes, including the L-phenylalanine catabolic process, oxylipin biosynthetic process, photosynthetic electron function, cell wall macromolecule catabolic process, or nitrate assimilation). However, the systemic signal at this time was not highly affected by the bacteria, and probably 24 h after treatment was not enough for significant changes to occur in the distal leaf.

#### 3.2.2. The GO Terms Most Abundantly Represented in the Plants Exposed to Larvae with Natural Intestinal Microflora

An enrichment analysis was performed to determine the 10 GO terms with the highest number of DEGs in each of the following categories: BP, MF, and CC.

**Figure 5:**
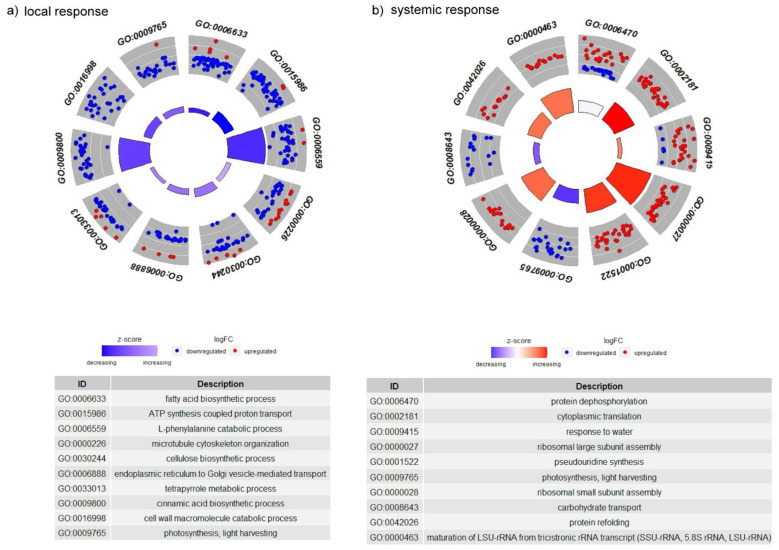
The 10 GO terms with the highest number of differentially expressed genes for the biological process category for (**a**) local and (**b**) systemic responses in the wheat plant leaves damaged by feeding of CLB larvae with natural bacterial flora (compared to those in the leaves wounded by CLB larvae with a reduced number of bacteria (as a control)).

In wheat leaves on which the CLB larvae with natural bacterial flora were feeding, the highest number of DEGs were related to the following GO terms in the BP category: fatty acid biosynthesis process, ATP synthesis coupled proton transport, microtubule cytoskeleton organization, cellulose biosynthetic process, endoplasmic reticulum to Golgi vesicle-mediated transport, tetrapyrrole metabolic process, cell wall microtubule catabolic process, photosynthesis, light-harvesting, cinnamic acid biosynthetic process, and L-phenylalanine catabolic process (Figure 5a). In the MF category, the GO terms represented by the highest number of DEGs were: ATP binding, structural constituent of the ribosome, and calcium ion binding (Figure S2a). Within the CC category, the highest number of DEGs were assigned, among others, to the GO terms: chloroplast thylakoid membrane, chloroplast stroma, chloroplast envelope, and photosystem II reaction centre (Figure S2b). The GO terms represented by the highest numbers of DEGs in the BP, MF, and CC categories were mostly associated with down-regulated DEGs.

In distal leaves, the presence of CLB larvae-associated bacteria resulted in the highest number of DEGs associated with the following GO terms representing the BP category: protein dephosphorylation, cytoplasmic translation, response to water, ribosomal large and small subunit assembly, pseudouridine synthesis, photosynthesis, light-harvesting, carbohydrate transport, protein refolding, and maturation of LSU–rRNA from tricistronic rRNA transcript (SSU-rRNA, 5.8S rRNA, and LSU-rRNA). Most of the above-mentioned processes (except photosynthesis, light-harvesting, and carbohydrate transport) were associated with a higher number of up- than down-regulated DEGs (Figure 5b). For the protein dephosphorylation process, the numbers of up- and down-regulated DEGs were similar. In the MF category, the most abundantly represented DEGs were related, among others, to the following GO terms: ATP binding, and structural constituents of the ribosome (Figure S3a). With regard to the CC category, the highest number of DEGs was associated, for instance, with chloroplast, large cytosolic, and small ribosomal subunits (Figure S3b). The GO terms of the MF and CC categories were associated with a higher number of up-regulated than down-regulated DEGs.

In both local and systemic responses, the GO terms: photosynthesis and light-harvesting (BP category) were mainly associated with down-regulated DEGs (Figure 5). Within the MF category, the following GO terms: ATP binding, structural constituent of the ribosome, GTPase activity, NAD binding, and pyridoxal phosphate binding, were associated with the highest numbers of DEGs. In the local response, the previously mentioned processes were associated with a higher number of down-regulated DEGs, in contrast to the systemic response, in which a higher number of up-regulated DEGs were noted (Figures S2a and S3a). Some of the most abundantly represented GO terms, including the L-phenylalanine catabolic process (local response), ribosomal large and small subunit assembly, pseudouridine synthesis, and maturation of LSU–rRNA from tricistronic rRNA transcript (SSU-rRNA, 5.8S rRNA, and LSU-rRNA) (systemic response), were also indicated as significant in the plant defense response to feeding of CLB larvae with the natural intestinal microflora.

#### 3.2.3. KEGGs Pathways Analysis

The KEGG database was used to examine which biological pathways in wheat plants were modulated by CLB-associated bacteria during the feeding of their insect host. We selected the biological pathways associated with the biological processes that have been indicated as significant for plant defense response to the feeding of the antibiotics-untreated CLB larvae. Up- and down-regulated DEGs were analysed together.

**Figure 6:**
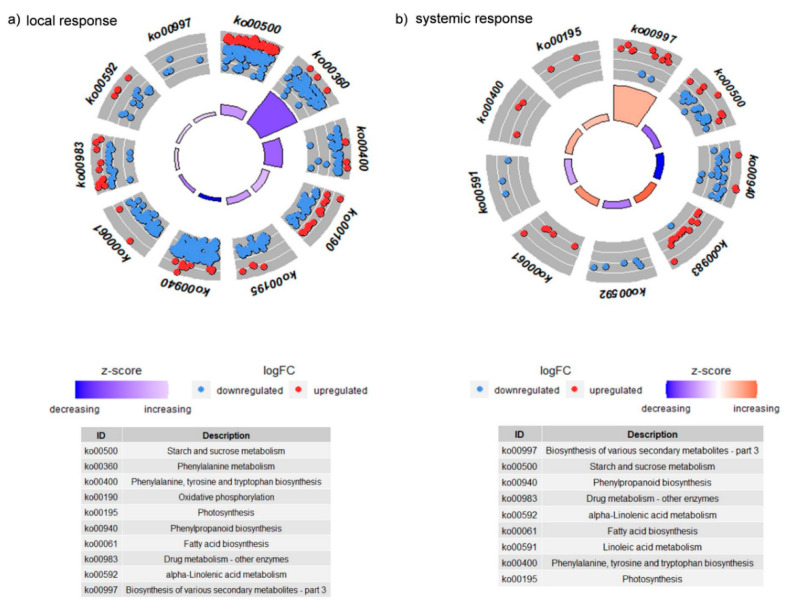
The transcripts distribution of plant metabolic pathways received after KEGG analysis, for (**a**) local and (**b**) systemic responses to the feeding of CLB larvae with natural bacterial flora, compared to the data obtained for the plants exposed to CLB larvae with a reduced number of bacteria (as a control).

In the wounded leaves of the plants exposed to the CLB larvae with the natural bacterial flora, the pathways related to phenylalanine metabolism (ko00360), and phenylpropanoid biosynthesis (ko00940) were associated with the highest number of transcripts, and most of them were down-regulated (Figure 6a and Table S8a).

In the distal leaves, the number of transcripts related to selected pathways was significantly lower compared to the results obtained from the larvae-damaged leaves (local response). Similarly, as in the local response, the pathway related to phenylpropanoid biosynthesis (ko00940) was also predominantly associated with down-regulated genes, whereas in the pathways related to the biosynthesis of various secondary metabolites (ko00997) and drug metabolism, the other enzymes (ko00983) were mainly associated with up-regulated transcripts, contrary to the results obtained for the wounded leaves (local response) (Figure 6b and Table S8b).

The obtained results indicate that the bacteria associated with the CLB larvae have an impact on many biological pathways, including those related to the synthesis of plant secondary metabolites upon CLB larvae feeding in both distal and wounded leaves.

#### 3.2.4. CLB Larvae-Associated Bacteria Modulate the Plant Signaling Pathways

**Figure 7:**
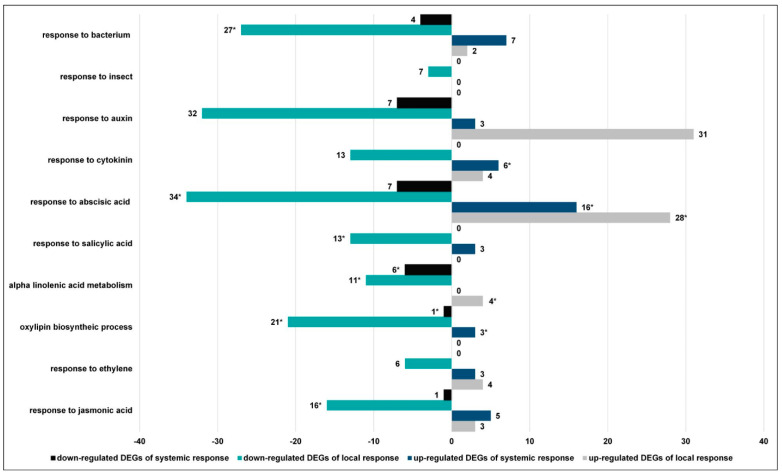
Differentially expressed genes (DEGs) distribution representing selected GO terms associated with plant signaling pathways in the wounded (local response) and distal leaves (systemic response) of the wheat plants exposed to the CLB larvae with natural bacterial flora (compared to the datasets obtained for the leaves wounded by larvae with a reduced number of bacteria (control)).* asterisk indicates an enriched process.

The objective of the next stage of our study was to determine if CLB larvae-associated bacteria have an impact on wheat plant signaling pathways upon CLB larvae feeding, manifested both in the local and systemic responses. As far as local response is concerned, in the plants wounded by antibiotics-untreated CLB larvae, the JA, SA, and cytokinin-dependent signaling pathways were associated with more down- than up-regulated DEGs. Within the abscisic acid (ABA), ethylene (ET), and auxin-dependent pathways, the numbers of up- and down-regulated transcripts were similar (Figure 7 and Table S9a). We found a lower expression level of the transcripts of the allene oxide cyclase (*AOC*), which is associated with response to some of the phytohormones mediated pathways, alpha-linolenic acid metabolism, and defense response both to insect and bacterium. Within the GO term of the oxylipin biosynthetic process, the genes encoding lipoxygenase (LOX) (including *TraesCS5A02G378900*, *TraesCS5B02G382600*, *TraesCS5D02G388800*, and *TraesCS5D02G388700*) were significantly down-regulated in the plants exposed to larvae with natural bacterial flora when compared to those in the plants wounded by the antibiotics-treated CLB larvae. For the SA-dependent pathway, the lower expression level of the gene encoding WRKY62 (*TraesCS5A02G225500*) was observed. The genes encoding pathogenesis-related protein 1-like (PR-1-like) are considered as markers of the SA-dependent pathway. However, their lower expression level within the ABA-activated signaling pathway was noted in the plants wounded by the antibiotics-untreated larvae (Table S9a).

As to the JA-dependent signaling pathway, the lower expression level of the genes encoding threonine, isoleucine, phenylalanine, tyrosine motif transcriptional factor 9 (TIFY9) and TIFY3, and an increase in the expression level of the genes encoding NINJA-the negative regulator of JA, were demonstrated in the plants exposed to the larvae with natural intestinal microflora. When the plants were wounded by the antibiotics-untreated CLB larvae, within the GO term of response to the bacterium, a lower expression of a number of genes was found, among others, those encoding WRKY62, NPR1 (nonexpressor of pathogenesis-related (PR) gene 1)-type protein 2, pathogenesis-related protein 4-like (PR-4-like), mitogen-activated protein kinase 3 (MAPK 3), or the aforementioned AOC (Table S9a).

In distal leaves, the number of DEGs associated with the analysed signaling pathways was significantly lower than in the wounded leaves. The aforementioned signaling pathways were predominantly associated with up-regulated DEGs except for the auxin-dependent pathways (Figure 7 and Table S9b). In the JA-dependent pathway, when the wheat plants were wounded by the CLB larvae with natural bacterial flora, increased expression of DEGs encoding IAA-amino acid hydrolase, DEAD-box ATP-dependent RNA helicase 50, and a lower expression level of gene encoding BTB/POZ domain and ankyrin repeat-containing protein NPR5-like (*TraesCS3B02G537400*) were observed. The lower expression level of *TraesCS3B02G537400* was also noted for the GO term of response to the bacterium. Within the GO term of the oxylipin biosynthetic process, a very large decrease in the expression of the gene encoding one lipoxygenase (*TraesCS5D02G041100*) was shown in the plants exposed to the untreated-antibiotics larvae (Table S9b).

#### 3.2.5. CLB Larvae-Associated Bacteria Modulate Processes Associated with the Synthesis of Antifeedant Proteins, Photosynthesis, and Plant Cell Wall Organization

Another objective of our study was also to establish if and, if yes, in which way the CLB larvae-associated bacteria modulate photosynthesis, plant cell wall organization, and synthesis of proteins that are potentially detrimental for insects.

**Figure 8:**
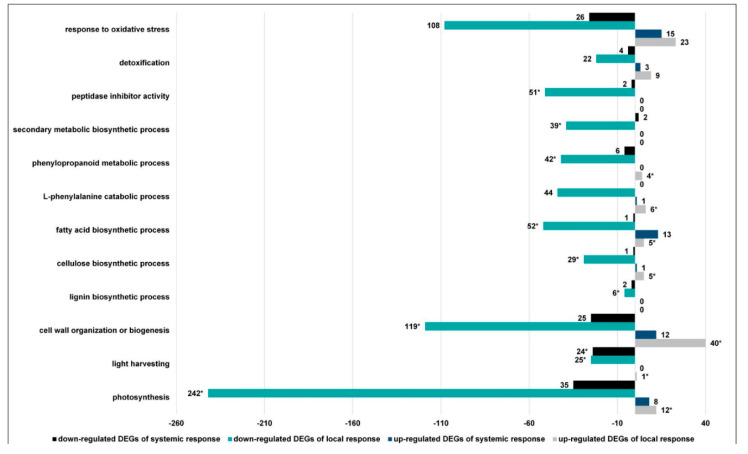
Differentially expressed genes (DEGs) distribution representing selected GO terms associated with photosynthesis, cell wall organization and synthesis of proteins, and metabolites that can have detrimental effects on CLB larvae in wounded (local response) and distal leaves (systemic response) of plants exposed to the CLB larvae with natural bacterial flora (in comparison to datasets obtained for the leaves wounded by larvae with a reduced number of bacteria (control)). * asterisk indicates an enriched process.

The transcriptome analysis performed for the wounded leaves showed that 24 h after larvae feeding initiation, the insect-associated bacteria played a key role in modulating the GO term related to the secondary metabolite biosynthesis process. This GO term was only associated with down-regulated DEGs (Figure 8). For instance, significantly lower levels of expression of genes encoding phenylalanine ammonia-lyase (PAL), cinnamyl alcohol dehydrogenase, transaldolase, and MAPK were recorded (Table S10a). The GO terms related to the phenylpropanoid metabolic process and L–phenylalanine catabolic process were predominantly associated with down-regulated DEGs (Figure 8), and a decrease in the expression levels of a number of genes encoding PAL was observed (Table S10a). The analysis also indicated a significantly lower expression of many genes encoding cysteine and serine protease inhibitors in the leaves damaged by the larvae with natural bacterial flora (Table S10a). The GO term of response to oxidative stress was also associated with more down-regulated than up-regulated DEGs (Figure 8), and a decrease in the expression of many DEGs, especially those encoding peroxidases, including *TraesCS2D02G107600*, *TraesCS3D02G305300*, and *TraesCS3A02G297200* was noted (Table S10a).

The bacteria associated with the CLB larvae had a significant impact on cell wall organization as well as photosynthetic and biogenesis processes (more down- than up-regulated DEGs were noted) (Figure 8). A decrease in the expression levels of a number of genes encoding, for instance, pectinesterase, expansin, chitinase, glucan synthase, or xylanase inhibitor was reported (Table S10a). The lignin and cellulose biosynthetic processes were predominantly associated with down-regulated DEGs, and a much lower expression of DEGs related to these processes was noted in the plants exposed to the larvae with natural bacterial flora. For instance, for the GO term of cellulose biosynthetic process, several genes encoding cellulose and glucan synthase were characterized with much lower levels of expression. The GO term of fatty acid biosynthetic process was also mainly associated with down-regulated DEGs and a decrease in the expression levels of, e.g., the genes encoding very long-chain 3-oxoacyl-CoA synthase, lipoxygenase or 3-ketoacyl-CoA synthase was observed in the leaves wounded by the CLB larvae untreated with antibiotics, compared to the data obtained for the leaves exposed to the antibiotics-treated larvae (Figure 8 and Table S10a).

In the distal leaves of the plants damaged by the CLB larvae with natural bacterial flora, only two DEGs (both down-regulated) were involved in the processes related to the synthesis of plant metabolites potentially detrimental to insects (Figure 8 and Table S10b). The phenylpropanoid metabolic process was only associated with down-regulated DEGs, and a decrease in the expression levels of, e.g., genes encoding laccase or cinnamoyl-CoA reductase was noted. The GO term, response to oxidative stress, was associated with more down-than up-regulated DEGs (Figure 8 and Table S10b). In the leaves on which the CLB larvae with natural bacterial flora were feeding, lower expression levels of the genes encoding peroxidases (especially *TraesCS2B02G124600* and *TraesCS2D02G107600*) were observed. The CLB larvae-associated bacteria have also impacted on a large decrease in the expression of genes encoding inhibitors of the serine protease family (*TraesCS4D02G298300* and *TraesCS4A02G005700*). In distal leaves of the plants exposed to the antibiotics-untreated larvae, the photosynthetic, light-harvesting, cell wall organization or biogenesis processes were mainly associated with down-regulated DEGs (Figure 8). Within the GO term of cell wall organization, and biogenesis, a decrease in the expression level, of, among others, the genes encoding pectinesterase, expansin, or xyloglucan endotransglucosylase/hydrolase was indicated. The fatty acid biosynthetic process was associated with more up- than down-regulated DEGs. Only the DEG encoding very long-chain (3R)-3-hydroxyacyl-CoA dehydratase was down-regulated (*TraesCS1B02G295000*) (Figure 8 and Table S10b).

#### 3.2.6. Genes Revealing the Opposite Direction of Expression in Response to Feeding of Larvae with Natural and Reduced Bacterial Flora

At the next stage of our study, we wanted to find out which genes of the GO terms within the BP category showed the opposite direction of expression in the plants on which the CLB larvae with natural bacterial flora were feeding, relative to the results obtained from the plants wounded by larvae with a reduced number of bacteria.

For the local response, the greatest opposite differences in expression levels were noted for the genes representing the GO terms related to cellulose biosynthetic process, cell wall organization, and biogenesis. For instance, the genes encoding glucan synthase (*TraesCS2B02G23650*, *TraesCS2D02G217100*, and *TraesCS2A02G210400*) were down-regulated in the plants wounded by the larvae with natural bacterial flora, in opposition to the data obtained for the plants wounded by the antibiotics-treated larvae, for which these genes were up-regulated (Table 1 and Table S11). Within the GO term related to response to oxidative stress, the genes encoding peroxidases (*TraesCS6B02G063800*, *TraesCS6D02G054800*, *TraesCS6A02G047500*, and *TraesCS6B02G063900*) were also down-regulated in the plants damaged by the CLB larvae with the natural intestinal flora, in contrast to their expression in the plants damaged by the antibiotics-treated larvae (Table 1 and Table S11).

#### 3.2.7. Validation of Transcriptome Data

The expression of selected DEGs was checked once again using RT-qPCR to validate the RNA-seq results. The direction of changes in gene expression observed on the basis of RT-qPCR data was consistent with that determined by RNA-seq (Figure S4).

## 4. Discussion

While feeding, herbivorous insects introduce components (including bacteria) of their oral secretion into plant tissue, which might affect the gene expression in damaged and distal plant parts [10,11,29,30]. The effect of CLB-associated bacteria on the overall defense response of the wheat plants on which CLB feeds has not yet been investigated. Chung et al. [16] have indicated that *Wolbachia* may play a key role in shaping the tomato plant’s defensive response to CPB feeding. We have previously indicated that at least 90% of the CLB populations tested are associated with *Wolbachia* and *Rickettsia* [12], which may suggest that the above-mentioned genera of bacteria may have a significant impact not only on the insect host but also on the plant defense response. Therefore, the main aim of this study was to check how the bacteria associated with the CLB larvae modulated the wheat’s response upon larvae feeding.

The greatest changes in DEGs distribution and their expression levels in the leaves wounded (local response) by the larvae (Figure 2) suggest that not only tissue damage but also insect-related factors had a significant effect on the plant defense response. The insect’s foraging causes a massive plant response, which may be due to the insect’s feeding mode or the presence of numerous components in its oral secretion. Differences between the defense responses to the feeding of untreated (B) and antibiotics-treated larvae (C) (much smaller number of DEGs in B than in C plant treatment) may suggest that the larvae-associated bacteria mitigate, to some extent, plant defense responses to larvae feeding at the analysed time point.

In natural conditions, plants are constantly exposed to multiple stressors, both abiotic and biotic (including bacteria, fungi, viruses, nematodes, or herbivorous insects) [8,31]. Because the plant’s defense response is associated with high energy cost, the plant needs to balance growth and defense to minimize damage and energy expenditure, which could have a negative impact on plant development, and the quality and quantity of crop yield. Plant threat recognition has a significant impact on the induction of proper signaling pathways, as well as on local and systemic expression of transcripts. A large body of research data have indicated that signaling pathway crosstalk plays a fundamental role in fine-tuning the growth-defense processes [32]. The JA, SA, and ET are central plant defense mediators, but cytokinins, as well as ABA and auxin-dependent pathways, play an important role in modulating plant response [33]. It is considered that the SA-dependent pathway is primarily induced in response to biotrophic pathogens [34] and herbivores causing slight tissue disruption (i.e., piercing-sucking), whereas the JA pathway is primarily activated by and involved in defense against insect herbivores, and together with ET, necrotrophic pathogens [32,34]. Through the modulation of JA signaling branches, ET and ABA control plant responses to herbivores [35].

According to our results, 24 h after the initiation of insect feeding, the CLB larvae-associated bacteria had an important effect on plant signaling pathways, especially at the sites of their insect host feeding. Namely, in the plants exposed to the CLB larvae with natural bacterial flora, the expression levels of most of the genes associated with ABA, SA, JA, ET, auxin, and cytokinin were lower in comparison to the data obtained for the plants wounded by the larvae with a reduced bacterial flora. For instance, a decrease in the expression of the genes encoding AOC involved in the synthesis of JA [36] was recorded.

According to the GO enrichment analysis, the oxylipin biosynthetic process was the most significant defense response in the leaves on which the larvae with natural bacterial flora were feeding. This process was associated only with down-regulated DEGs. For instance, a much lower expression level of, among others, the genes encoding LOX was noted in the plants exposed to larvae with natural bacterial flora, compared to that obtained for the plants damaged by antibiotics-treated larvae. The most common substrates for LOXs are linolenic and linolenic acids [37]. As follows from the transcriptomic analyses, in the plants on which the CLB larvae with natural bacterial flora were feeding, the GO term related to alpha-linolenic acid was associated with a higher number of down- (including DEGs encoding AOC) than up-regulated DEGs. Concerning the JA-dependent signaling pathway, the higher expression level of the genes encoding the NINJA-negative regulator of JA was noted in the plants damaged by the larvae with natural bacterial flora. It is known that the genes encoding NINJA proteins repress the transcription factors that regulate the expression of JA-responsive genes [35,38]. Interestingly, in the leaves on which the antibiotics-untreated larvae were feeding, within defense response to bacterium process, a lower expression level of, among others, the transcripts encoding WRKY62, NPR1-type protein 2, PR-4-like, MAPK 3, and the aforementioned AOC were found, in comparison to the data obtained from the plants wounded by antibiotics-treated larvae.

Members of the PR-1 family are among the most abundantly produced proteins in plants upon pathogen attack and play an antimicrobial function. PR-1 gene expression has been used as a marker for the SA-dependent signaling pathway [39]. The transcriptomic analyses showed a lower expression level of a number of genes encoding PR-1 in the leaves on which larvae with natural intestinal microflora were feeding, compared to the data obtained for those wounded by larvae with a reduced number of bacteria. Lower expression of the genes encoding PR-1 may suggest that CLB larvae-associated bacteria can also influence the SA-dependent signaling pathway.

It is known that herbivorous insects can take advantage of the natural cross-talk between hormonal pathways to circumvent plant defenses [35]. Some of them can suppress several plant signaling pathways. For instance, *Tetranychus evansi* represses both JA and SA-dependent signaling pathways in tomatoes, which results in a decrease in defense-related components [35,40]. Meanwhile, *Bemisia tabaci* activates SA-dependent response and suppresses JA-dependent defense response, which improves whitefly performance [35,41]. There is growing evidence that insect-associated microbes are important players in manipulating plant response to the benefit of the insect host [16]. For instance, it was indicated that endosymbiotic bacteria *Wolbachia* can change plant physiology, which has a positive effect on the leaf miners’ (*Phyllonorycter blancardella*, Lepidoptera, Gracillariidae) performance. *Wolbachia* induced a green-island phenotype in apple leaves, preserving photosynthetically active tissues in senescent leaves. In turn, larvae of *Scaptomyza flava* (Diptera, Drosophilidae) vectors *Pseudomonas syringae* bacteria to and from the feeding site. Larvae perform better on plants infected with *P. syringae*, which suggests that these bacteria suppress antiherbivore defense [14,42].

A plant responds to wounding by a variety of defense mechanisms, including fortification of cell walls, induction of defense-related genes, synthesis of antimicrobial and anti-nutritional compounds, or the initiation of processes leading to wound healing. Several pathways, including the phenylpropanoid one, act as major sources of plant defense metabolites, including those with anti-herbivore properties, which can affect insect growth and development [34]. The transcriptomic analyses showed that 24 h after the initiation of larvae feeding, the CLB-associated bacteria had a significant impact on anti-herbivore defense processes and pathways. For instance, the KEGGs analyses indicated that in the leaves on which the CLB larvae with natural bacterial flora were feeding, among others, the pathway related to phenylpropanoid biosynthesis was mostly associated with down-regulated DEGs. In addition, within the GO term related to phenylpropanoid biosynthesis lower expression levels of a number of genes encoding PAL (known for its involvement in the conversion of primary metabolites to secondary metabolites) [43] were noted in the plants exposed to larvae with natural bacterial flora, compared to those obtained for the plants wounded by antibiotics-treated insects. Interestingly, KEGGs pathway analysis indicated also that in the wounded leaves, the pathway related to the biosynthesis of various secondary metabolites was mainly associated with down-regulated DEGs, contrary to the results obtained for distal leaves. The lower expression of many genes associated with the above-mentioned pathways in the plants exposed to the CLB larvae with natural bacterial flora, compared to the data obtained for the plants wounded by antibiotics-treated larvae, may indicate that the bacteria had a significant impact on the pathways responsible for the synthesis of a number of metabolites potentially harmful to herbivorous insects.

It is assumed that the JA-dependent pathway is related to the production of insecticidal and antifeedant proteins, which can reduce herbivore development and survival [44]. For instance, enzyme inhibitors can cause different types of deleterious effects when ingested by insects [45]. Thus they are considered important elements of natural plant defense and comprise one of the most abundant classes of proteins in plants [45]. Plant protease inhibitors not only regulate the activity of plant proteases but also exogenous proteases of insects, bacteria, and fungi to limit cellular damage [46]. Inhibitors interact with proteolytic enzymes, reducing or neutralizing their catalytic activity [45]. As a result, the amount of available amino acids is reduced, which can impair insect growth and reproductive potential and development [45]. Thus, plant protease inhibitors may have a cumulative protective effect on plants. Our previous study was focused on understanding the expression of genes encoding cysteine and serine protease inhibitors in wheat plants wounded by CLB larvae with natural intestinal microflora and reduced bacterial components [19]. The transcriptomic analysis provided much more information on this topic and revealed that many genes encoding inhibitors belonging to the cysteine and serine protease inhibitor families showed a significantly lower level of expression in the leaves damaged by larvae with natural bacterial flora, compared to the data obtained for the leaves damaged by larvae with a reduced number of bacteria. This situation can be beneficial for CLB larvae, which, among others, contain cysteine and serine proteases [47].

The insects’ nutrition can also be disrupted by several enzymes, including peroxidases, that impair the nutrient uptake by insects [48,49,50,51]. Production of phenoxy and other oxidative radicals by the peroxidases in association with phenols directly deters the feeding of insects and/or produces toxins that reduce the plant’s digestibility, which in turn leads to nutrient deficiency in insects with a drastic effect on their growth and development. In addition, peroxidase direct toxicity in the gut of herbivores was indicated [48,52,53]. The transcriptomic analysis of wheat plants exposed to the CLB larvae showed that CLB larvae-associated bacteria had an impact on the GO term related to the local and systemic response to oxidative stress, probably to the benefit of their insect host. This supposition is supported by the fact that this GO term was associated with more down-than up-regulated DEGs, and significantly lower expression of a number of genes, especially those encoding peroxidases, was reported in the plants wounded by the larvae with natural intestinal microflora, compared to the data obtained for the plants exposed to the larvae with a reduced number of bacteria. The larvae-associated bacteria were also found to switch the direction of expression of some genes encoding peroxidases (*TraesCS6B02G063800*, *TraesCS6D02G054800*, *TraesCS6A02G047500*, and *TraesCS6B02G063900*), because their down-regulation was noted in the plants damaged by the antibiotics-untreated larvae, in contrast to their up-regulation in the plants damaged by the antibiotics-treated larvae.

Plant cell expansion requires both continuous deposition and modification of the cell wall [54]. Increasing the leaf toughness reduces the feeding by herbivores and also decreases the nutritional content of the leaf [48]. It has been shown that the accumulation of lignin enhances the plant’s resistance against aphids. Cinnamoyl–CoA reductases and cinnamyl alcohol dehydrogenases are involved in lignin synthesis [48]. In our study, in the leaves on which the CLB larvae with natural intestinal microflora were feeding, lower expression levels of a number of genes related to the lignin and cellulose biosynthetic processes, including those encoding cinnamyl alcohol dehydrogenases, cellulose, and glucan synthase, were observed.

According to our results, in the wounded and distal leaves of the plants exposed to larvae with natural bacterial flora, the GO terms of photosynthesis and light-harvesting were associated with more down- than up-regulated DEGs. The CLB larvae feeding disturbs the photosynthetic machinery not only at the site of feeding but also in distal parts of the plant. It has been reported that various pathogens and herbivores’ activities result in disruption of the photosynthetic machinery, loss of photosynthetic tissue, and/or disruption of the vasculature, affecting water and sugar transport which has a negative impact on photosynthesis [32,55,56].

Our transcriptomic analyses showed that 24 h after the initiation of larvae feeding, the CLB-associated bacteria influenced the expression levels of many genes and, in some cases, reversed the direction of changes in the expression of certain genes, including those related to protein phosphorylation and dephosphorylation, photosynthesis, oxidative stress response, or cell wall organization and biogenesis. The CLB-associated bacteria may be involved in regulating the early immune response of wheat plants following CLB larvae feeding because it was indicated that they had a significant impact on many plant defense processes and pathways. It was indicated that in the leaves on which the antibiotics-untreated larvae were feeding, the pathways related to the biosynthesis of various secondary metabolites (including the phenylpropanoid pathway) were mainly associated with down-regulated DEGs, contrary to the results obtained for distal leaves. The larvae-associated bacteria were also shown to modulate the plant signaling pathways and affect the expression of genes associated with the JA-signaling pathway, e.g., a decrease in the expression level of several genes encoding protease inhibitors was noted in the plants wounded by the larvae with natural bacterial flora. Many studies have indicated that the insect gut should be the target for insect control and that plant protease inhibitors can be very effective tools for insect control. However, protease inhibitors may not be fully effective because the insects have developed, over time, different mechanisms to mitigate the effects of the inhibitors, and insect-associated bacteria can contribute to their digestive processes.

## 5. Conclusions

In response to the analysed plant treatments in the wounded leaves, significantly greater changes (at a *p* value < 0.05) in gene expression took place than in the distal leaves. A much larger number of up-and down-regulated DEGs were reported in damaged leaves than in distal ones, depending on the plant treatment. The differences in DEGs distribution in the leaves exposed to the antibiotics-treated (7751 up- and 5769 down-regulated DEGs) and non-treated larvae (3822 up- and 2756 down-regulated DEGs) may indicate that the plant’s response was significantly influenced by the bacteria associated with the insect, and that in the leaves on which antibiotics-treated larvae were feeding greater changes in the expression levels of genes associated with metabolic pathways took place.

According to the obtained results, CLB larvae-associated bacteria modulate processes associated with the synthesis of antifeedant proteins (including protease inhibitors), photosynthesis, and plant cell wall organization and signaling pathways, indicating that those processes play a crucial role in *T. aestivum* response to *O. melanopus* in this three-way interaction model. Additionally, we observed that the insect-associated bacteria can reverse the direction of changes in the expression of certain genes, among others, those encoding glucan synthases or peroxidases. The midgut of the insect should be the target of their control due to the presence of bacteria and enzymes that are crucial for the proper functioning of the insects. Understanding the role of insect-associated bacteria in the plant’s defense response might be useful in developing effective tools for insect control in the future and contributing to the expansion of knowledge on the plant host and herbivorous insect ecology and evolution.

## Figures and Tables

**Table 1:** Genes of selected biological processes with the opposite directions of expression in the larval-damaged plants with natural bacterial flora, compared to those in the larval-damaged plants with a reduced number of bacteria, for local and systemic responses.

**Local Response**					
**Up-Regulated DEGs in Response to CLB with Natural** **Bacterial Flora** **Down-Regulated DEGs in Response to CLB with a Reduced** **Bacteria Content**			**Down-Regulated DEGs in Response to CLB with Natural Bacterial Flora** **Up-Regulated DEGs in Response to CLB with a Reduced Bacteria Content**		
**GO ID**	Go Name	# Seq	GO ID	Go Name	# Seq
**GO:0006470**	protein dephosphorylation	5	GO:0071554	cell wall organization or biogenesis	6
**GO:0009737**	response to abscisic acid	2	GO:0006979	response to oxidative stress	4
			GO:0015979	photosynthesis	4
			GO:0030244	cellulose biosynthetic process	3
**Systemic Response**					
**GO:0006470**	protein dephosphorylation	2			

## Data Availability

The original contributions presented in the study are included in the article/Supplementary Material, further inquiries can be directed to the corresponding author. The datasets (sequencing reads) used and/or analysed during the current study are deposited in the National Center for Biotechnology Information (SRA database) under the BioProject number PRJNA793396.

## Supplementary Materials

The following supporting information can be downloaded at: https://www.mdpi.com/article/10.3390/cells11152342/s1, Table S1: Primers sequences used for RT-qPCR amplification of the seven differentially expressed genes selected for validation. Table S2: A number of common differentially expressed genes in response to mechanical damage (A), wounding caused by CLB larvae with natural bacterial flora (B), and by CLB larvae with a reduced number of bacteria (C) in wounded and distal leaves; Table S3: A number of unique differentially expressed genes for local and systemic plant response to mechanical damage (A), feeding of CLB larvae with natural bacterial flora (B), and feeding of CLB larvae with a reduced number of bacteria (C); Table S4: GO enrichment terms of biological process category shared the largest number of common differentially expressed genes (up- and down-regulated) in wheat leaves wounded (local response) mechanically (A), by feeding of CLB larvae with natural bacterial flora (B), and by larvae with a reduced number of bacteria (C); Table S5: GO enrichment terms of biological process category for unique up- and down-regulated differentially expressed genes in wheat leaves damaged (local response) mechanically (A), or by feeding of CLB larvae with natural bacterial flora (B), and by feeding of larvae with a reduced number of bacteria (C); Table S6: GO enrichment terms of biological process category counting the largest number of common differentially expressed genes (up- and down-regulated) in distal leaves (systemic response) of wheat plants wounded mechanically (A), by feeding CLB larvae with natural bacterial flora (B), and by larvae with a reduced number of bacteria (C); Table S7: GO enrichment terms of biological process category for unique up- and down-regulated differentially expressed genes in distal leaves (systemic response) of wheat plants damaged mechanically (A), or by feeding of CLB larvae with natural bacterial flora (B), and by feeding of larvae with a reduced number of bacteria (C); Figure S1: A number of up-and down-regulated differentially expressed genes in wounded (local response) and distal leaves (systemic response) of plants exposed to larvae with natural bacterial flora, in comparison to the dataset obtained for the leaves wounded by larvae with a reduced number of bacteria; Figure S2: The 10 GO terms with the highest number of differentially expressed genes for the (a) molecular function and (b) cellular component category in wheat leaves (local response) on which CLB larvae with natural bacterial flora were feeding (compared to those in the plant leaves wounded by CLB larvae with a reduced number of bacteria (as a control)); Figure S3: The 10 GO terms with the highest number of differentially expressed genes for the (a) molecular function and (b) cellular component category in distal leaves (systemic response) of wheat plant damaged by feeding of CLB larvae with natural bacterial flora (compared to those in plants wounded by CLB larvae with a reduced number of bacteria (as a control)); Figure S4: The expression level (logFC) of seven selected differentially expressed genes (DEGs) in wounded (local response) and distal leaves (systemic response) of wheat plants exposed to CLB larvae with natural bacterial flora (compared to those of plants damaged by CLB larvae with a reduced number of bacteria, as controls). * asterisks indicate the statistical significance of the results: * *p* < 0.05, Mann–Whitney test. POX-peroxidase, GS-probable mixed-linked glucan synthase 3, CPI-cysteine proteinase inhibitor, LOX-lipoxygenase, AOC-allene-oxide cyclase, NPR-protein NRT1/PTR FAMILY 6.2, CAD-cell division protein; Table S8: Transcripts associated with selected pathways in (a) wounded (local response), (b) distal leaves (systemic response) in plants on which CLB larvae with natural bacterial flora were feeding compared to data obtained from plants damaged by CLB larvae with a reduced bacterial flora (treated as a control); Table S9: Differentially expressed genes (DEGs) distribution representing selected GO terms associated with plant signaling pathways in (a) the wounded (local response) and (b) distal leaves (systemic response) of the wheat plants exposed to the CLB larvae with natural bacterial flora, compared to data obtained from plants damaged by larvae with a reduced number of bacteria (treated as a control); Table S10: Distributions of differentially expressed genes (DEGs) representing selected GO terms associated with photosynthesis, cell wall organization, and synthesis of proteins, metabolites that can have a detrimental effect on CLB larvae in (a) wounded (local response) and distal leaves (systemic response) of plants exposed to the CLB larvae with natural bacterial flora, compared to data obtained for plants damaged by larvae with a reduced number of bacteria (treated as a control); Table S11: Differentially expressed genes (DEGs) of selected biological processes with the opposite directions of expression in the larval-damaged plants with natural bacterial flora, compared to those in the larval-damaged plants with a reduced number of bacteria, for local and systemic responses.
