# Supplementary material for: The Impact of Oulema melanopus—Associated Bacteria on the Wheat Defense Response to the Feeding of Their Insect Hosts
Source: Cells. 2022 Jul 29;11(15):2342. doi: 10.3390/cells11152342 (PMC9367625; doi:10.3390/cells11152342)
Supplement: Supplementary file 1 [file cells-11-02342-s001.zip › cells-1828543 proof sup/cells-1828543-XML supplementary.pdf]

Supplementary Material

The Impact of *Oulema melanopus*–Associated Bacteria on the Wheat Defense Response to the Feeding of Their Insect Hosts

Beata Wielkopolan[1], Patryk Frąckowiak[2], Przemysław Wieczorek[2], Aleksandra Obrępalska-Stępłowska[2*]

[1] Department of Monitoring and Signaling of Agrophages, Institute of Plant Protection – a National Research Institute, 60-318 Poznań, Poland; B.Wielkopolan@iorpib.poznan.pl (B.W.)
[2] Department of Molecular Biology and Biotechnology, Institute of Plant Protection – a National Research Institute, 60-318 Poznań, Poland; P.Frackowiak@iorpib.poznan.pl (P.F.); P.Wieczorek@iorpib.poznan.pl (P.W.)
* Correspondence: olaob@o2.pl (A.O.-S.)

Table S1. Primers sequences used for RT-qPCR amplification of the seven differentially expressed genes selected for validation.

**Gene ID**	**ID name**	**Sequences of primers**
TraesCS6D02G162800	Cinnamyl alcohol dehydrogenase	F:5′GCAAGTCCTCCCAAATCCAC-3′<br>R:5′AGGTCCTGTTTTCCTGAGGG-3′
TraesCS4B02G037900	Lipoxygenase	F:5′CCTCACACAGCTCACACAAC-3′<br>R:5′TCCAGCACGTTCTTCCTCAT-3′
TraesCS3A02G331500	Cysteine proteinase inhibitor	F:5′GTCGTGCCGTTTACTCAGAC-3′<br>R:5′ATTGGCCTTGTTGTTGTGCT-3′
TraesCS1B02G038700	Protein NRT1/ PTR FAMILY 6.2	F:5′CTACCTCACCATCGCCATCT-3′<br>R:5′GATGAGGTAGAGGCACACGT-3′
TraesCS6B02G365200	Allene-oxide cyclase	F:5′GCTACGAGGCCATCTACAGC-3′<br>R:5′AAGGGGAAGACGATCTGGTT-3′
TraesCS2B02G236500	Probable mixed-linked glucan synthase 3	F:5′CAACCTCAACTTCGCCAACA-3′<br>R:5′CACCTTGCCGTTGATCTAGC-3′
TraesCS6B02G063800	Peroxidase	F:5′ATTGCTTGCTTGCCCTCTTC-3′<br>R:5′AAGCTACCCACGTCATCCAA-3′

Ta54227 [54]	Cell division protein	F:5’CAGCTGCTGACTGAGATGGA-3’<br>R:5’ATGTCTGGCCTGTTGGTAGC-3’

Table S2. A number of common differentially expressed genes in response to mechanical damage **(A)**, wounding caused by CLB larvae with natural bacterial flora **(B)**, and by CLB larvae with a reduced number of bacteria **(C)** in wounded and distal leaves.

Response	Experimental variant	No of common genes
local (wounded leaves)	A+B+C	1150
	B+C	968
	A+B	436
	A+C	2065
systemic (distal leaves)	A+B+C	16
	B+C	49
	A+B	64
	A+C	114

Table S3. A number of unique differentially expressed genes for local and systemic plant response to mechanical damage **(A)**, feeding of CLB larvae with natural bacterial flora **(B)**, and feeding of CLB larvae with a reduced number of bacteria **(C)**.

Response	Experimental treatments	No of unique genes
local (wounded leaves)	(A)	859
	(B)	4024
	(C)	9337
systemic (distal leaves)	(A)	1672
	(B)	1132
	(C)	876

Table S4. GO enrichment terms of biological process category shared the largest number of common differentially expressed genes (up- and down-regulated) in wheat leaves wounded (local response) mechanically **(A)**, by feeding of CLB larvae with natural bacterial flora **(B)**, and by larvae with a reduced number of bacteria **(C)**.

	A+B+C				B+C			
	GO ID	GO Name	p vaule	nr. of transcripts	GO ID	GO Name	p vaule	nr. of transcripts
up-regulated	GO:0008610	lipid biosynthetic process	0,003368	70	GO:0009408	response to heat	6,97E-09	10
	GO:0006869	lipid transport	1,31E-13	22	GO:0006096	glycolytic process	4,06E-06	8
	GO:0030244	cellulose biosynthetic process	2,84E-07	18	GO:0046686	response to cadmium ion	4,73E-07	7
	GO:0006096	glycolytic process	3,31E-09	14	GO:0042026	protein refolding	1,24E-07	6
	GO:0042742	defense response to bacterium	1,29E-06	14	GO:0009651	response to salt stress	0,001653	6
	GO:0009834	plant-type secondary cell wall biogenesis	0,002961	14	GO:0006564	L-serine biosynthetic process	1,66E-08	5
	GO:0050832	defense response to fungus	0,000182	9	GO:0016042	lipid catabolic process	0,008491	5
	GO:0009086	methionine biosynthetic process	3,82E-06	6	GO:0006260	DNA replication	0,022959	5
	GO:0009738	abscisic acid-activated signaling pathway	9,3E-05	6	GO:0042546	cell wall biogenesis	0,027502	5
	GO:0002181	cytoplasmic translation	0,000245	6	GO:0055129	L-proline biosynthetic process	2,44E-07	4
	GO:0010497	plasmodesmata-mediated intercellular transport	0,000728	6	GO:0006086	acetyl-CoA biosynthetic process from pyruvate	3,45E-06	4
	GO:0019510	S-adenosylhomocysteine catabolic process	0,017461	5	GO:0061077	chaperone-mediated protein folding	4,88E-05	4
	GO:1901671	positive regulation of superoxide dismutase activity	3,94E-05	4	GO:0000413	protein peptidyl-prolyl isomerization	0,001934	4
	GO:0046274	lignin catabolic process	0,005131	4	GO:0042742	defense response to bacterium	0,012155	4
	GO:0009408	response to heat	0,005381	4	GO:0010446	response to alkaline pH	1,46E-07	3
down-regulated	GO:0120009	intermembrane lipid transfer	6,72E-05	2	GO:0006470	protein dephosphorylation	0,006391	5
	GO:0045736	negative regulation of cyclin-dependent protein serine/threonine kinase activity	0,000603	1	GO:0007166	cell surface receptor signaling pathway	0,000339	4
	GO:1904263	positive regulation of TORC1 signaling	0,005418	1	GO:0045893	positive regulation of transcription, DNA-templated	0,030623	4
	GO:0061408	positive regulation of transcription from RNA polymerase II promoter in response to heat stress	0,007218	1	GO:0009113	purine nucleobase biosynthetic process	2,1E-05	3
	GO:0035672	oligopeptide transmembrane transport	0,007817	1	GO:0071586	CAAX-box protein processing	0,000115	3
	GO:0009772	photosynthetic electron transport in photosystem II	0,012598	1	GO:0016554	cytidine to uridine editing	0,000162	3
	GO:0046856	phosphatidylinositol dephosphorylation	0,033834	1	GO:0009116	nucleoside metabolic process	0,002799	3
	GO:0009411	response to UV	0,036748	1	GO:0030244	cellulose biosynthetic process	0,004075	3
	GO:0000724	double-strand break repair via homologous recombination	0,04255	1	GO:0090229	negative regulation of red or far-red light signaling pathway	7,99E-06	2
	-	-	-	-	GO:0080006	internode patterning	7,99E-06	2
	-	-	-	-	GO:1904263	positive regulation of TORC1 signaling	0,000284	2
	-	-	-	-	GO:0032196	transposition	0,000284	2
	-	-	-	-	GO:0048236	plant-type sporogenesis	0,000611	2
	-	-	-	-	GO:0009788	negative regulation of abscisic acid-activated signaling pathway	0,001324	2
	-	-	-	-	GO:0006189	'de novo' IMP biosynthetic process	0,003748	2

	A+B				A+C			
	GO ID	GO Name	p vaule	nr. of transcripts	GO ID	GO Name	p vaule	nr. of transcripts
up-regulated	GO:0009415	response to water	8,18E-05	8	GO:0006979	response to oxidative stress	3,19E-10	48
	GO:0001522	pseudouridine synthesis	0,000199	4	GO:0042744	hydrogen peroxide catabolic process	8,39E-11	40
	GO:0006826	iron ion transport	0,000499	4	GO:0006633	fatty acid biosynthetic process	3,97E-09	24
	GO:1900865	chloroplast RNA modification	1,07E-05	3	GO:0006559	L-phenylalanine catabolic process	1,51E-17	18
	GO:0016554	cytidine to uridine editing	0,000467	3	GO:0009735	response to cytokinin	3,64E-10	14
	GO:0042026	protein refolding	0,00078	3	GO:0009800	cinnamic acid biosynthetic process	4,13E-14	13
	GO:0042273	ribosomal large subunit biogenesis	0,010956	3	GO:0098869	cellular oxidant detoxification	2,02E-05	13
	GO:0030036	actin cytoskeleton organization	0,039739	3	GO:0009767	photosynthetic electron transport chain	1,96E-05	12
	GO:0110102	ribulose bisphosphate carboxylase complex assembly	1,65E-05	2	GO:0009409	response to cold	0,000271	11
	GO:0006427	histidyl-tRNA aminoacylation	0,000342	2	GO:0000470	maturation of LSU-rRNA	8,4E-09	10
	GO:1901259	chloroplast rRNA processing	0,001452	2	GO:0006032	chitin catabolic process	2,37E-06	10
	GO:0010089	xylem development	0,001452	2	GO:0050832	defense response to fungus	0,001597	10
	GO:0000077	DNA damage checkpoint signaling	0,003942	2	GO:0000278	mitotic cell cycle	0,007666	10
	GO:0010311	lateral root formation	0,004289	2	GO:0006228	UTP biosynthetic process	1,45E-12	9
	GO:0009263	deoxyribonucleotide biosynthetic process	0,007091	2	GO:0006183	GTP biosynthetic process	1,45E-12	9
down-regulated	GO:0015979	photosynthesis	0,000219	4	GO:0045944	positive regulation of transcription by RNA polymerase II	0,000187	6
	GO:0016042	lipid catabolic process	0,000134	3	GO:0000398	mRNA splicing, via spliceosome	0,012705	4
	GO:0010112	regulation of systemic acquired resistance	4,68E-05	2	GO:0006680	glucosylceramide catabolic process	1,51E-05	3
	GO:0042773	ATP synthesis coupled electron transport	0,047574	1	GO:0034968	histone lysine methylation	0,003828	3
	GO:0030026	cellular manganese ion homeostasis	0,017898	1	GO:0042545	cell wall modification	0,006731	3
	-	-	-	-	GO:0045003	double-strand break repair via synthesis-dependent strand annealing	0,000192	2
	-	-	-	-	GO:0051091	positive regulation of DNA-binding transcription factor activity	0,000255	2
	-	-	-	-	GO:0009934	regulation of meristem structural organization	0,000408	2
	-	-	-	-	GO:0000086	G2/M transition of mitotic cell cycle	0,001076	2
	-	-	-	-	GO:0007020	microtubule nucleation	0,002047	2
	-	-	-	-	GO:0005978	glycogen biosynthetic process	0,003793	2
	-	-	-	-	GO:0019252	starch biosynthetic process	0,006346	2
	-	-	-	-	GO:0046856	phosphatidylinositol dephosphorylation	0,013187	2
	-	-	-	-	GO:0008643	carbohydrate transport	0,047558	2
	-	-	-	-	GO:0016444	somatic cell DNA recombination	0,006078	1

Table S5. GO enrichment terms of biological process category for unique up- and down-regulated differentially expressed genes in wheat leaves damaged (local response) mechanically **(A)**, or by feeding of CLB larvae with natural bacterial flora **(B)**, and by feeding of larvae with a reduced number of bacteria **(C)**.

	GO ID	GO Name	P-Value	Nr of transcripts		GO ID	GO Name	P-Value	Nr of transcripts
**Mechanical Damage (A)**									
up-regulated	GO:0009627	systemic acquired resistance	2,46061E-09	9	down-regulated	GO:0006355	regulation of transcription, DNA-templated	0,005704415	17
	GO:0030026	cellular manganese ion homeostasis	1,19008E-05	5		GO:0000160	phosphorelay signal transduction system	2,86E-03	4
	GO:0010020	chloroplast fission	1,78855E-05	4		GO:0006183	GTP biosynthetic process	3,33952E-06	3
	GO:0034755	iron ion transmembrane transport	2,13455E-05	4		GO:0006228	UTP biosynthetic process	3,33952E-06	3
	GO:0001522	pseudouridine synthesis	0,001434036	4		GO:0010112	regulation of systemic acquired resistance	1,47E-05	3
	GO:0042273	ribosomal large subunit biogenesis	0,007748055	4		GO:0006241	CTP biosynthetic process	5,98936E-05	3
	GO:0006261	DNA-dependent DNA replication	0,00981844	4		GO:0006004	fucose metabolic process	0,000655351	3
	GO:0015698	inorganic anion transport	0,026434442	4		GO:0008643	carbohydrate transport	0,001530463	3
	GO:0042372	phylloquinone biosynthetic process	0,000112369	3		GO:0006165	nucleoside diphosphate phosphorylation	0,006149768	3
	GO:0006995	cellular response to nitrogen starvation	0,000139741	3		GO:0006729	tetrahydrobiopterin biosynthetic process	0,000171305	2
	GO:0006542	glutamine biosynthetic process	0,000139741	3		GO:0046621	negative regulation of organ growth	0,000513056	2
	GO:0042793	plastid transcription	0,00024678	3		GO:0034968	histone lysine methylation	0,017422996	2
	GO:0048510	regulation of timing of transition from vegetative to reproductive phase	0,000396083	3		GO:0010411	xyloglucan metabolic process	0,033856213	2
	GO:0000373	Group II intron splicing	0,000593562	3		GO:0090307	mitotic spindle assembly	0,003931715	1
	GO:0048564	photosystem I assembly	0,000844714	3		GO:0003352	regulation of cilium movement	0,005891811	1
**CLB larvae with natural bacterial flora (B)**									
	**GO ID**	**GO Name**	**P-Value**	**Nr of transcripts**		**GO ID**	**GO Name**	**P-Value**	**Nr of transcripts**
up-regulated	GO:0006470	protein dephosphorylation	1,42309E-05	20	own-regulated	GO:0006468	protein phosphorylation	3,28043E-31	262
	GO:0045893	positive regulation of transcription, DNA-templated	0,037690238	12		GO:0071555	cell wall organization	4,10E-02	23
	GO:0006457	protein folding	0,034628137	11		GO:0048544	recognition of pollen	0,014726261	18
	GO:0006260	DNA replication	0,02649369	10		GO:0006887	exocytosis	4,52694E-05	15
	GO:0016554	cytidine to uridine editing	5,93066E-09	9		GO:0007015	actin filament organization	1,46E-03	11
	GO:0009793	embryo development ending in seed dormancy	0,003550539	9		GO:0007166	cell surface receptor signaling pathway	0,000319225	10
	GO:0009631	cold acclimation	1,04201E-08	8		GO:0005992	trehalose biosynthetic process	4,7541E-06	9
	GO:0008643	carbohydrate transport	0,00054524	8		GO:0030026	cellular manganese ion homeostasis	5,16681E-06	8
	GO:0009408	response to heat	0,0026669	8		GO:0016998	cell wall macromolecule catabolic process	0,001330354	8
	GO:0006777	Mo-molybdopterin cofactor biosynthetic process	7,65381E-07	6		GO:0030244	cellulose biosynthetic process	0,004260453	8
	GO:0045492	xylan biosynthetic process	0,000105963	6		GO:1901002	positive regulation of response to salt stress	0,000109028	6
	GO:0006415	translational termination	0,000134275	6		GO:1900150	regulation of defense response to fungus	0,000318159	6
	GO:0005985	sucrose metabolic process	0,000377441	6		GO:0006032	chitin catabolic process	0,00892505	6
	GO:0006383	transcription by RNA polymerase III	0,000500298	5		GO:0046373	L-arabinose metabolic process	4,11955E-05	5
	GO:0009742	brassinosteroid mediated signaling pathway	0,001634218	5		GO:0000165	MAPK cascade	7,17639E-05	5
**CLB larvae with a reduced number of bacteria (C)**									
	**GO ID**	**GO Name**	**P-Value**	**Nr of transcripts**		**GO ID**	**GO Name**	**P-Value**	**Nr of transcripts**
up-regulated	GO:0045454	cell redox homeostasis	1,10341E-05	41	down-regulated	GO:0048544	recognition of pollen	0,016528096	29
	GO:0006096	glycolytic process	3,08565E-10	31		GO:0006486	protein glycosylation	1,70E-02	25
	GO:0006633	fatty acid biosynthetic process	6,82835E-05	31		GO:0045944	positive regulation of transcription by RNA polymerase II	0,022180646	19
	GO:0015986	ATP synthesis coupled proton transport	5,3385E-09	30		GO:0017004	cytochrome complex assembly	3,8774E-08	17
	GO:0009651	response to salt stress	1,51071E-05	28		GO:0015886	heme transport	2,77E-12	16
	GO:0006457	protein folding	0,021439455	27		GO:0009734	auxin-activated signaling pathway	0,012291396	15
	GO:0006888	endoplasmic reticulum to Golgi vesicle-mediated transport	7,70666E-10	25		GO:0036297	interstrand cross-link repair	2,94321E-06	9
	GO:0006890	retrograde vesicle-mediated transport, Golgi to endoplasmic reticulum	8,64486E-16	24		GO:0046470	phosphatidylcholine metabolic process	0,001970065	8
	GO:0009611	response to wounding	0,001978288	24		GO:0005985	sucrose metabolic process	0,003480262	8
	GO:0031408	oxylipin biosynthetic process	6,86066E-20	22		GO:0000724	double-strand break repair via homologous recombination	0,01156812	8
	GO:0009737	response to abscisic acid	0,021125225	22		GO:0010228	vegetative to reproductive phase transition of meristem	0,036474925	8
	GO:0046686	response to cadmium ion	7,67606E-09	20		GO:0009785	blue light signaling pathway	2,08811E-06	7
	GO:0006099	tricarboxylic acid cycle	1,07198E-07	20		GO:0006298	mismatch repair	0,001009518	7
	GO:0032482	Rab protein signal transduction	1,79352E-13	18		GO:0010119	regulation of stomatal movement	0,00118544	7
	GO:0006559	L-phenylalanine catabolic process	1,29397E-10	18		GO:0009245	lipid A biosynthetic process	7,97212E-06	6

Table S6. GO enrichment terms of biological process category counting the largest number of common differentially expressed genes (up- and down-regulated) in distal leaves (systemic response) of wheat plants wounded mechanically **(A)**, by feeding CLB larvae with natural bacterial flora **(B)**, and by larvae with a reduced number of bacteria **(C)**.

	A+B+C				B+C			
	GO ID	GO Name	p vaule	nr. of transcripts	GO ID	GO Name	p vaule	nr. of transcripts
up-regulated	GO:0006468	protein phosphorylation	0,007809	4	-	-	-	-
up-regulated	GO:0106035	protein maturation by [4Fe-4S] cluster transfer	3,41E-07	2	-	-	-	-
up-regulated	GO:0048544	recognition of pollen	0,002149	2	-	-	-	-
up-regulated	GO:0070588	calcium ion transmembrane transport	0,007374	1	-	-	-	-
up-regulated	GO:0016226	iron-sulfur cluster assembly	0,009717	1	-	-	-	-
down-regulated	-	-	-	-	GO:0015977	carbon fixation	2E-08	3
down-regulated	-	-	-	-	GO:0006099	tricarboxylic acid cycle	2E-07	3
down-regulated	-	-	-	-	GO:0006355	regulation of transcription, DNA-templated	0,0122	3
down-regulated	-	-	-	-	GO:0006809	nitric oxide biosynthetic process	0,0011	1
down-regulated	-	-	-	-	GO:0042128	nitrate assimilation	0,002	1

	A+B				A+C			
	GO ID	GO Name	p vaule	nr. of transcripts	GO ID	GO Name	p vaule	nr. of transcripts
up-regulated	GO:0071555	cell wall organization	0,000291	4	GO:0006468	protein phosphorylation	0,0199	6
up-regulated	GO:0042183	formate catabolic process	1,93E-09	3	GO:0006096	glycolytic process	5E-05	3
up-regulated	GO:0006811	ion transport	0,040056	3	GO:0048544	recognition of pollen	0,0008	3
up-regulated	GO:0006886	intracellular protein transport	0,049923	2	GO:0048268	clathrin coat assembly	0,0001	2
up-regulated	GO:0032482	Rab protein signal transduction	0,000176	2	-	-	-	-
up-regulated	GO:0006353	DNA-templated transcription, termination	0,008468	1	-	-	-	-
up-regulated	GO:0006378	mRNA polyadenylation	0,015005	1	-	-	-	-
up-regulated	GO:0000387	spliceosomal snRNP assembly	0,016866	1	-	-	-	-
down-regulated	GO:0006284	base-excision repair	0,002556	1	-	-	-	-
down-regulated	GO:0006470	protein dephosphorylation	0,015972	1	-	-	-	-
down-regulated	GO:0006289	nucleotide-excision repair	0,002831	1	-	-	-	-
down-regulated	-	-	-	-	-	-	-	-
down-regulated	-	-	-	-	-	-	-	-

Table S7. GO enrichment terms of biological process category for unique up- and down-regulated differentially expressed genes in distal leaves (systemic response) of wheat plants damaged mechanically **(A)**, or by feeding of CLB larvae with natural bacterial flora **(B)**, and by feeding of larvae with a reduced number of bacteria **(C)**.

Mechanical Damage (A)

up-regulated				down-regulated			
**GO ID**	**GO Name**	**P-Value**	**Nr of transcripts**	**GO ID**	**GO Name**	**P-Value**	**Nr of transcripts**
GO:0006468	protein phosphorylation	0,003368425	70	GO:0016042	lipid catabolic process	0,003324	6
GO:0006633	fatty acid biosynthetic process	1,31397E-13	22	GO:0045944	positive regulation of transcription by RNA polymerase II	6,25E-03	6
GO:0048544	recognition of pollen	2,83578E-07	18	GO:2000113	negative regulation of cellular macromolecule biosynthetic process	0,032972	6
GO:0000226	microtubule cytoskeleton organization	3,30914E-09	14	GO:0035024	negative regulation of Rho protein signal transduction	2,23E-07	3
GO:0006869	lipid transport	1,28886E-06	14	GO:0090630	activation of GTPase activity	8,87E-07	3
GO:0042744	hydrogen peroxide catabolic process	0,002961347	14	GO:0009865	pollen tube adhesion	8,87E-07	3
GO:0000278	mitotic cell cycle	0,000182297	9	GO:0009234	menaquinone biosynthetic process	4,39E-06	3
GO:0042542	response to hydrogen peroxide	3,82319E-06	6	GO:0009102	biotin biosynthetic process	1,22E-05	3
GO:0006032	chitin catabolic process	9,29656E-05	6	GO:0010218	response to far red light	0,000118	3
GO:0016998	cell wall macromolecule catabolic process	0,000245265	6	GO:0010099	regulation of photomorphogenesis	0,000142	3
GO:0071805	potassium ion transmembrane transport	0,000728191	6	GO:0010114	response to red light	0,000142	3
GO:0009409	response to cold	0,01746066	5	GO:0009860	pollen tube growth	0,000201	3
GO:0006833	water transport	3,93751E-05	4	GO:0010017	red or far-red light signaling pathway	0,000522	3
GO:0006081	cellular aldehyde metabolic process	0,005131101	4	GO:0015693	magnesium ion transport	0,00137	3
GO:0006914	autophagy	0,00538091	4	GO:0009846	pollen germination	0,00137	3

CLB larvae with natural bacterial flora (B)

up-regulated				down-regulated			
**GO ID**	**GO Name**	**P-Value**	**Nr of transcripts**	**GO ID**	**GO Name**	**P-Value**	**Nr of transcripts**
GO:0006357	gulation of transcription by RNA polymerase	0,030562648	7	GO:0042546	cell wall biogenesis	5,4E-07	12
GO:0009082	anched-chain amino acid biosynthetic proce	1,36075E-07	6	GO:0071555	cell wall organization	8,08E-04	12
GO:0006260	DNA replication	0,00988467	6	GO:0010411	xyloglucan metabolic process	2,5E-07	9
GO:0009631	cold acclimation	6,29705E-07	5	GO:0006535	cysteine biosynthetic process from serine	2,36E-07	6
GO:0000028	ribosomal small subunit assembly	7,57186E-07	5	GO:0048544	recognition of pollen	4,91E-02	6
GO:0000027	ribosomal large subunit assembly	1,21942E-05	5	GO:0006809	nitric oxide biosynthetic process	8,57E-10	5
GO:0006564	L-serine biosynthetic process	2,09838E-06	4	GO:0042128	nitrate assimilation	4,05E-08	5
GO:0006086	etyl-CoA biosynthetic process from pyruva	5,48202E-06	4	GO:0009630	gravitropism	0,000392	4
GO:0009561	megagametogenesis	4,35596E-05	4	GO:0019354	siroheme biosynthetic process	2,03E-07	3
GO:0031408	oxylipin biosynthetic process	6,89998E-05	4	GO:0010044	response to aluminum ion	8,08E-07	3
GO:0030490	maturation of SSU-rRNA	7,67571E-05	4	GO:0010929	positive regulation of auxin mediated signaling pathway	4,01E-06	3
GO:1990542	mitochondrial transmembrane transport	0,000305152	4	GO:0009236	cobalamin biosynthetic process	2,36E-05	3
GO:0001522	pseudouridine synthesis	0,000821138	4	GO:0000103	sulfate assimilation	0,00013	3
GO:0016114	terpenoid biosynthetic process	0,009696056	4	GO:0080113	regulation of seed growth	0,000183	3
GO:0034975	protein folding in endoplasmic reticulum	4,08756E-06	3	GO:0009788	negative regulation of abscisic acid-activated signaling pathway	0,000183	3

CLB larvae with a reduced number of bacteria (C)

up-regulated				down-regulated			
**GO ID**	**GO Name**	**P-Value**	**Nr of transcripts**	**GO ID**	**GO Name**	**P-Value**	**Nr of transcripts**
GO:0006629	lipid metabolic process	0,028129285	10	GO:0006470	protein dephosphorylation	3,66E-07	14
GO:0016180	snRNA processing	2,62701E-08	4	GO:0006869	lipid transport	6,79E-04	8
GO:0022900	electron transport chain	0,029082418	4	GO:0006730	one-carbon metabolic process	4,39E-09	7
GO:0035434	copper ion transmembrane transport	4,27975E-06	3	GO:0009086	methionine biosynthetic process	2,4E-07	6
GO:0006814	sodium ion transport	7,86984E-05	3	GO:0045454	cell redox homeostasis	4,15E-02	6
GO:0051301	cell division	0,004901227	3	GO:0006656	phosphatidylcholine biosynthetic process	4,04E-09	5
GO:0007059	chromosome segregation	0,00666272	3	GO:0009627	systemic acquired resistance	0,000102	5
GO:0007018	microtubule-based movement	0,00847582	3	GO:0019510	S-adenosylhomocysteine catabolic process	2,17E-08	4
GO:0006334	nucleosome assembly	0,021828096	3	GO:0006556	S-adenosylmethionine biosynthetic process	1E-06	4
GO:0000160	phosphorelay signal transduction system	0,038053652	3	GO:0016104	triterpenoid biosynthetic process	8,22E-05	4
GO:0006148	inosine catabolic process	6,18005E-06	2	GO:0042026	protein refolding	0,000163	4
GO:0006552	leucine catabolic process	3,69585E-05	2	GO:0030488	tRNA methylation	0,000249	4
GO:0050482	arachidonic acid secretion	0,000635187	2	GO:0010588	cotyledon vascular tissue pattern formation	9,45E-07	3
GO:0051560	mitochondrial calcium ion homeostasis	0,000921013	2	GO:0019464	glycine decarboxylation via glycine cleavage system	4,68E-06	3
GO:0009646	response to absence of light	0,00102768	2	GO:0010305	leaf vascular tissue pattern formation	1,94E-05	3

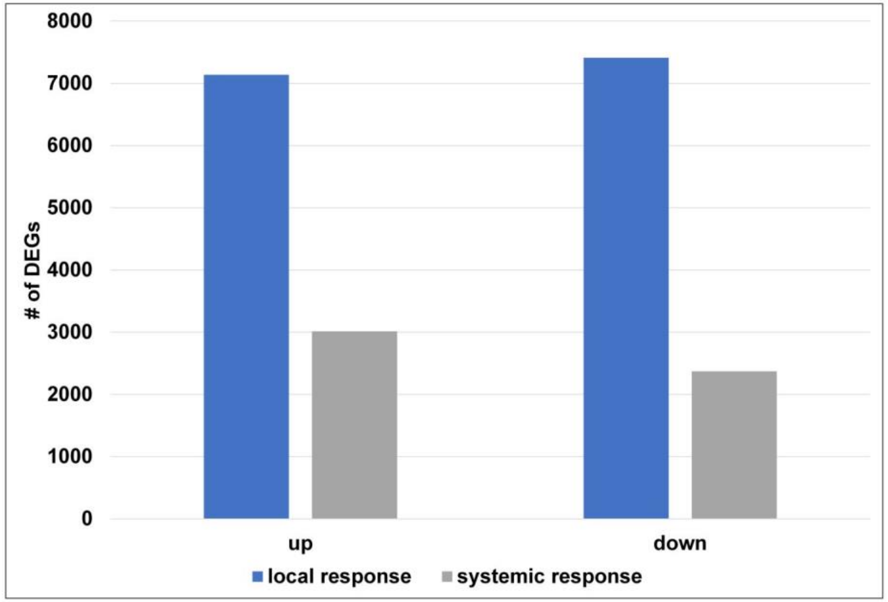

Figure S1. A number of up-and down-regulated differentially expressed genes (DEGs) in wounded (local response) and distal leaves (systemic response) of plants exposed to larvae with natural bacterial flora, in comparison to the dataset obtained for the leaves wounded by larvae with a reduced number of bacteria.

a) Molecular Function

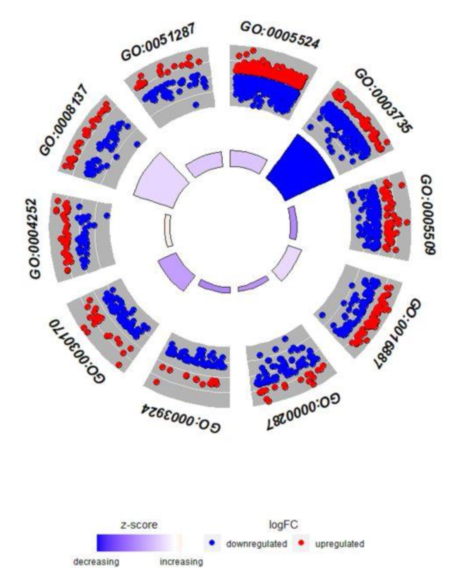

ID	Description
GO:0005524	ATP binding
GO:0003735	structural constituent of ribosome
GO:0005509	calcium ion binding
GO:0016887	ATP hydrolysis activity
GO:0000287	magnesium ion binding
GO:0003924	GTPase activity
GO:0030170	pyridoxal phosphate binding
GO:0004252	serine-type endopeptidase activity
GO:0008137	NADH dehydrogenase (ubiquinone) activity
GO:0051287	NAD binding

b) Cellular Component

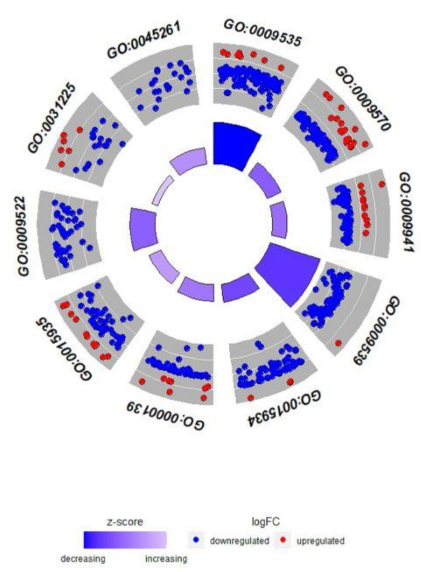

ID	Description
GO:0009535	chloroplast thylakoid membrane
GO:0009570	chloroplast stroma
GO:0009941	chloroplast envelope
GO:0009539	photosystem II reaction center
GO:0015934	large ribosomal subunit
GO:0000139	Golgi membrane
GO:0015935	small ribosomal subunit
GO:0009522	photosystem I
GO:0031225	anchored component of membrane
GO:0045261	proton-transporting ATP synthase complex, catalytic core F(1)

Figure S2. The 10 GO terms with the highest number of differentially expressed genes for the **a)** molecular function and **b)** cellular component category in wheat leaves (local response) on which CLB larvae with natural bacterial flora were feeding (compared to those in the leaves plants wounded by CLB larvae with a reduced number of bacteria (as a control)).

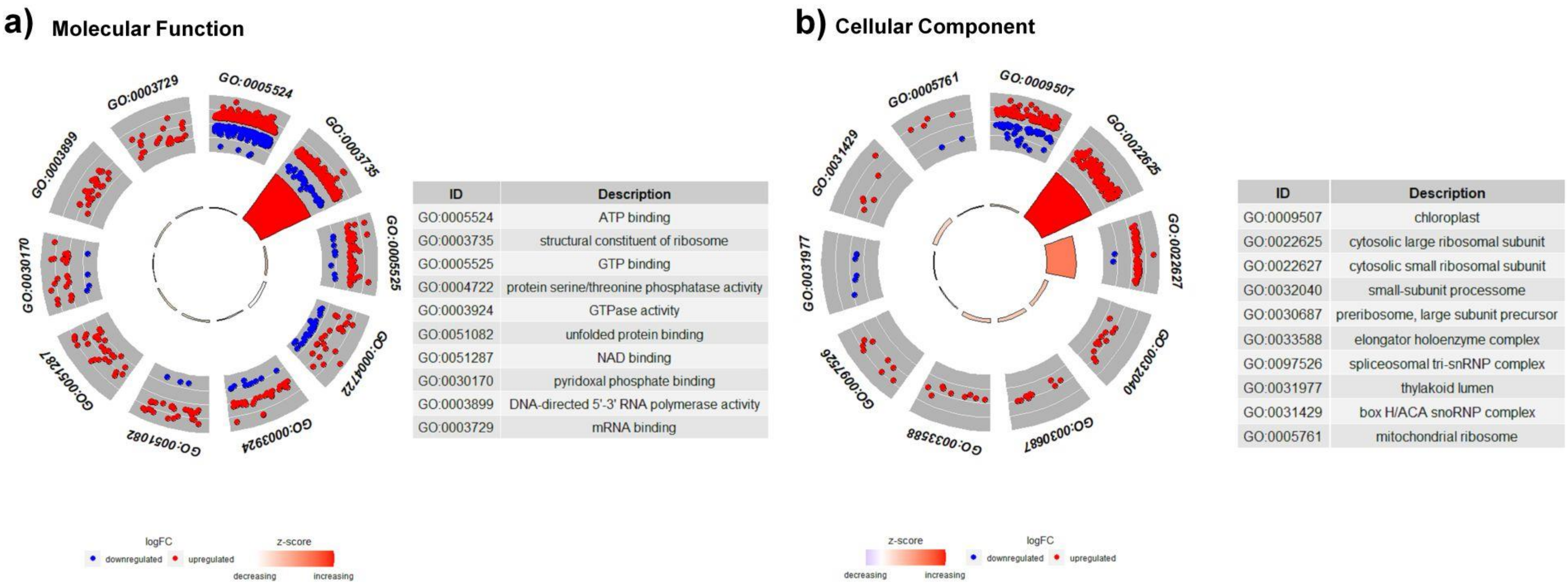

Figure S3. The 10 GO terms with the highest number of differentially expressed genes for the **a)** molecular function and **b)** cellular component category in distal leaves (systemic response) of wheat plant damaged by feeding of CLB larvae with natural bacterial flora (compared to those in plants wounded by CLB larvae with a reduced number of bacteria (as a control)).

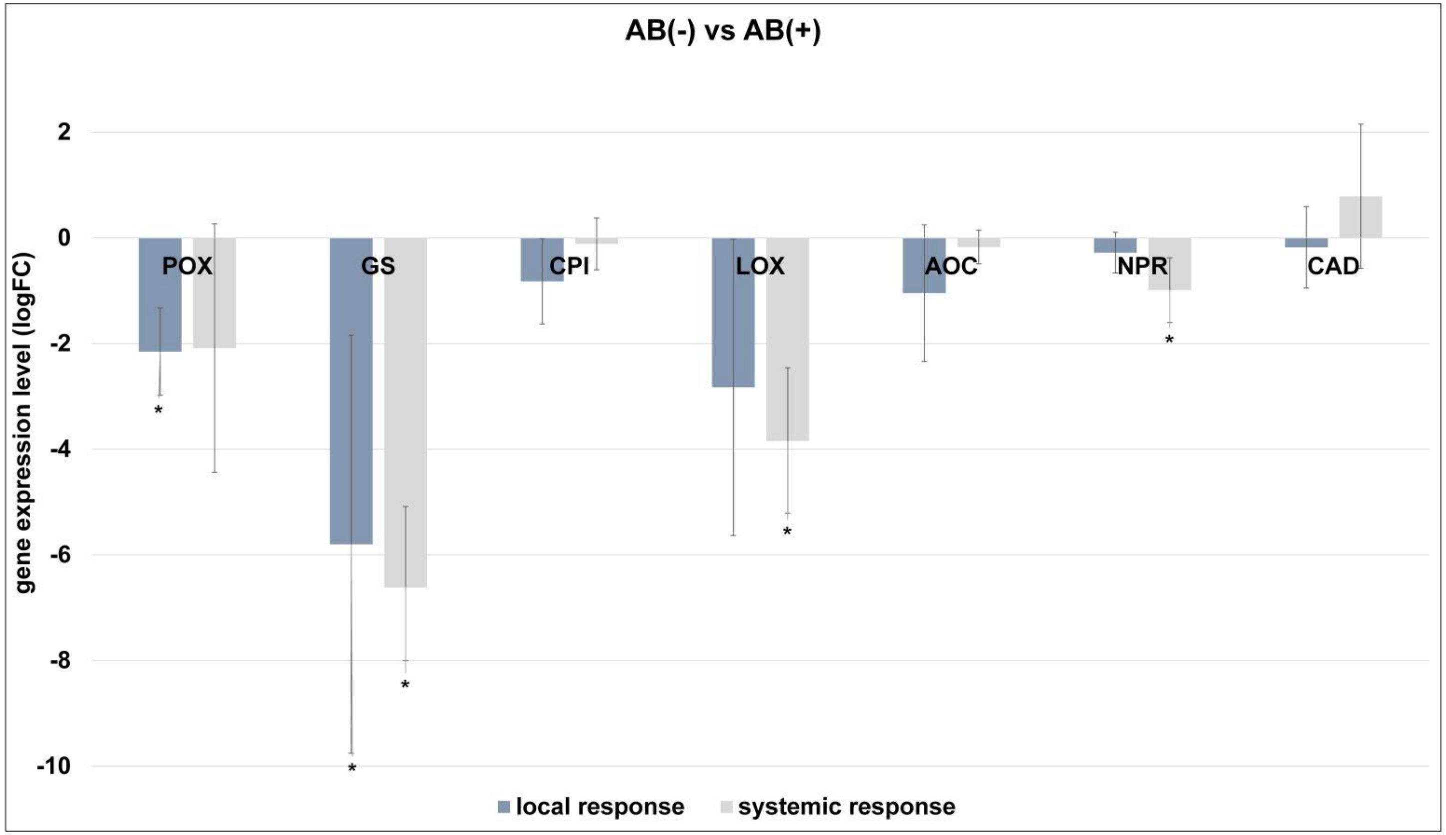

		POX	GS	CPI	LOX	AOC	NPR	CAD
	response/DEGs	*TraesCS6B02G063800*	*TraesCS2B02G236500*	*TraesCS3A02G331500*	*TraesCS4B02G037900*	*TraesCS6B02G365200*	*TraesCS1B02G038700*	*TraesCS6D02G162800*
RT-PCR	local response	-2,15294*	-5,79947*	-0,8248	-2,82872	-1,04658	-0,28013	-0,17939
	systemic response	-2,08639	-6,6184*	-0,11352	-3,84349*	-0,17371	-0,98922*	0,78754
RNA-seq	local response	-9,536465626*	-15,44216681*	-2,004*	-3,498*	-2,515*	-2,073*	-1,776*
	systemic response	-1,873323921	-2,629645678	0,122626596	-0,598303347	0,426301715	-0,680736542	1,224089764

Figure S4. The expression level (logFC) of seven selected differentially expressed genes (DEGs) in wounded (local response) and distal leaves (systemic response) of wheat plants exposed to CLB larvae with natural bacterial flora (compared to those of plants damaged by CLB larvae with a reduced number of bacteria, as controls). *asterisks indicate the statistical significance of the

results: * $p<0.05$, Mann-Whitney test. POX-peroxidase, GS-probable mixed-linked glucan synthase 3, CPI-cysteine proteinase inhibitor, LOX-lipoxygenase, AOC-allene-oxide cyclase, NPR-protein NRT1/PTR FAMILY 6.2, CAD-cell division protein.
